# Tangermeme: a toolkit for understanding *cis-*regulatory logic using deep learning models

**DOI:** 10.1038/s41592-026-03254-z

**Published:** 2026-10-08

**Authors:** Jacob Schreiber

**Affiliations:** 1 Research Institute of Molecular Pathology, Vienna BioCenter, Vienna, Austria; 2 Department of Genomics and Computational Biology, UMass Chan Medical School, Worcester, MA USA

**Keywords:** Machine learning, Software, Genomics

## Abstract

Deep learning models have achieved state-of-the-art performance at predicting diverse genomic modalities, yet their promise for biological discovery lies in how they are used after demonstrating their predictive performance. Here we describe the functionality of tangermeme, a highly optimized toolkit for ‘everything-but-the-model’ when it comes to genomic deep learning, and demonstrate how tangermeme can be used to distill the learned *cis-*regulatory patterns from models into human-interpretable insights.

## Abstract (web summary)

The tangermeme software package is a comprehensive, flexible and efficient Swiss Army knife for deep learning-based *cis*-regulatory pattern identification and analysis.

## Main

Machine learning models, such as hidden Markov models^1^ and support vector machines^2,3^, have been used in genomics for decades^4^. However, the rapid adoption of deep learning has shifted how machine learning is applied to genomic data^5–8^. A key strength of deep learning models in genomics is their end-to-end nature, where manual feature engineering is replaced by training directly on nucleotide sequence. Today, models can predict transcription factor (TF) binding^9^, histone modification^10^, chromatin accessibility^11–13^ and architecture^14,15^, transcription^16,17^, alternative splicing^18,19^, miRNA binding^20^, and RNA degredation rates^21^, including at single-cell or spatial resolution.

Although most attention has been paid to maximizing predictive performance, this is only the first step toward biological discovery^22^. After demonstrating accuracy, models can estimate the effects of noncoding variants to identify potential drivers^23,24^, be paired with feature attribution methods to identify nucleotides/motifs driving predictions^25,26^, be probed to identify global *cis-*regulatory rules^27,28^ or be used to design sequences with desired characteristics^29–31^. Crucially, this downstream usage is usually agnostic to model architecture or training; estimating variant effects is largely unaffected by whether a model uses convolutions or transformers, or by optimizer choice or learning rate.

Despite this modularity, an optimized repository for such methods that generalizes across model types does not yet exist. Instead, bespoke analysis code is typically packaged with models on release. Some software packages, such as Captum^32^, implement one or a few related algorithms. Other packages such as Selene^33^, kipoi^34^, CREsted^35^, EUGENe^36^ and gReLU^37^ focus on training and fine-tuning, with some even including model zoos of pretrained models. The ability to train or adapt models to new settings is invaluable, but support for using these models after training is more limited.

**Fig. 1:**
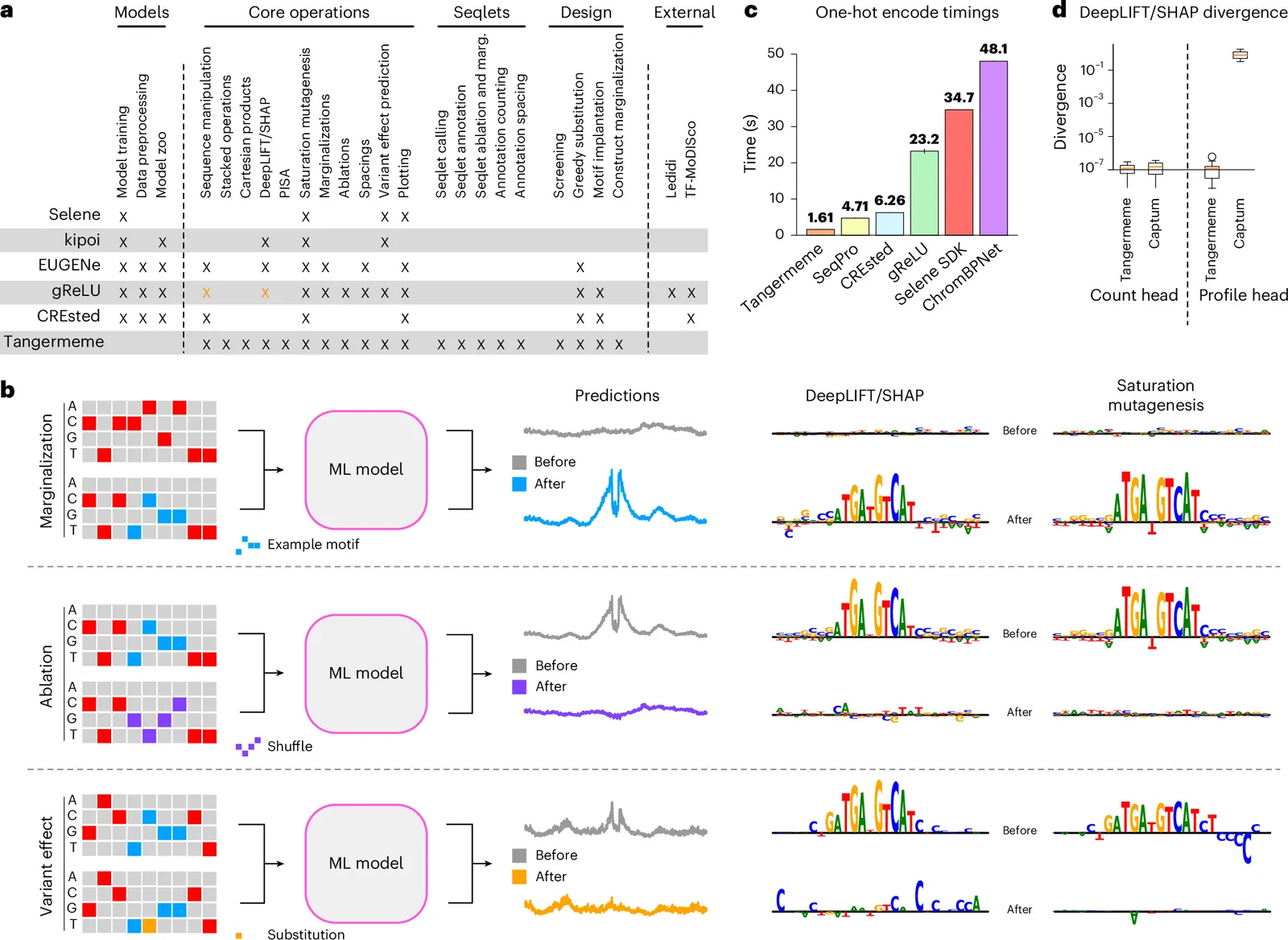
An overview of the tangermeme software suite. **a**, A comparison of functionality divided into support for model repositories and training (models), the implemented genomic analyses (core operations), support for seqlet calling and usage (seqlets), support for biological design (design) and built-in integration for external methods with mature codebases (external). Orange Xs indicate functionality that uses tangermeme. **b**, A cartoon schematic and simulation results demonstrating that sequence manipulations (the rows) can be separated by any model operation (the columns) and what the expected results might be when marginalizing a motif that the model responds to, ablating out a motif that the model responds to and introducing a variant that disrupts the motif. **c**, Timings for one-hot encoding hg38 chr1, averaged across ten runs. Medians and standard errors are plotted but, except for gReLU, are too small to easily see. **d**, Covergence deltas from 20 runs of DeepLIFT/SHAP in two settings with box plots showing medians as orange lines and quartiles and whiskers showing a maximum of the most extreme point or 1.5 times the interquartile range. With tangermeme, the custom profile head operation is provided to the function and so correctly registered, whereas Captum does not easily allow this.

To fill this niche, we introduce tangermeme, a software package that implements ‘everything-but-the-model’ for genomic deep learning. This design choice is intentional, as the computational operations within models and their training strategies are more diverse and rapidly evolving than their downstream use. Instead, tangermeme focuses on making the use of these trained models as simple as possible. Accordingly, it has easy-to-use modular implementations of sequence manipulations, to create relevant inputs, and operations involving models, such as efficiently making predictions or calculating feature attributions (Fig. 1a). This design philosophy leaves the details of loading and defining models to the user so as to not get locked in to any particular repository or the design choices made by others as to how models are stored and distributed.

A consequence of this modular design is that sequence manipulations and model operations can be stacked to create useful analyses (Fig. 1b). In silico marginalizations compare predictions before and after substituting a (usually short) sequence into a template, and thus can quickly identify relevant motifs from a database; ablations are the conceptual opposite and compare predictions before and after altering a region in the input that the model may be responding to. Variant effect estimations compare predictions before and after making one or a small number of (usually) noncontiguous substitutions and so can fine-map candidates to identify drivers. Importantly, prediction does not have to follow sequence manipulation; any model operation, including DeepLIFT/SHAP, in silico saturation mutagenesis or any custom operation, can be performed after sequence manipulation. This modularity enables researchers to easily create bespoke analyses for their specific research question. The influence of context on TF binding can be studied by ablating the nucleotides surrounding a known motif; the suitability of a regulatory element for use in a synthetic construct can be estimated by marginalizing the entire regulatory element; teasing apart the individual and joint effects of variants at the same locus can be done in a small number of commands.

Tangermeme supports multiple DNA design methods, with many being described by De Winter, Konstantakos and Aerts^31^. The simplest method is screening, where sequences are randomly generated, scored according to a user-defined objective function and the best is returned. Greedy substitution (sometimes called ‘directed evolution’) involves iteratively updating a randomly generated sequence by considering all possible single-nucleotide variants and choosing the single best one at each stage. Motif implantation proceeds similarly to greedy substitution except that entire motifs from a user-provided database are considered instead of individual mutations. Finally, a novel construct marginalization method conceptually merges in silico marginalization and design. Here, the task is to create short constructs that yield specified changes in model predictions on average across many background sequences. This procedure can be used to design libraries of inserts for constructs that induce desired activity, for example, patterns of chromatin accessibility, without needing to know anything about the construct.

For the same reason that tangermeme does not have built-in support for model repositories, it also avoids duplicating effort by wrapping existing implementations. For example, even though Ledidi^29^ and TF-MoDISco^38^ overlap with tangermeme’s design and attribution code, the existing code is widely used and maintained by others. Rather than ensuring ongoing compatibility with future updates to these codebases, even when their design choices are not aligned with those in tangermeme, we instead seek to have tangermeme be compatible with them so as to lower the burden of their future development. This choice is similar to how there is overlap between numpy’s^39^ linear algebra code and scikit-learn’s^40^ linear models, but numpy seeks to be the foundation for many packages.

Each of tangermeme’s operations are designed to simplify the practical usage of deep learning in genomics. When appropriate, operations have built-in batching, support across all data types and devices allowable in PyTorch and work out of the box on multi-input/output models. Each operation is low level and easily extended or integrated into research code. For instance, most DeepLIFT/SHAP implementations require all background sequences in graphics processing unit memory simultaneously, making massive models difficult to interpret. Tangermeme’s built-in batching removes this requirement without compromising exactness, and low-precision support further reduces the burden. Further, tangermeme can support models with any type of internal computational units, for example, convolutions, long short-term memory models, transformers and custom operations, as well as any type of prediction, for example, a single binary value or an entire profile. These models can operate on sequences of any length, limited only by needing to have the central processing unit/graphics processing unit memory to run a single such sequence through the model. Finally, core operations are faster than other software. It takes tangermeme less than 2 s to one-hot encode the entirety of hg38 chr1 (Fig. 1c)—approximately three times faster than the next fastest implementation. Although one-hot encoding is conceptually simple, being this fast enables on-the-fly batch generation and is reflective of the optimizations taken across tangermeme.

By implementing these algorithms abstractly and validating them, we identified underappreciated issues in other commonly used codebases. DeepLIFT/SHAP is widely used for feature attribution and involves overriding the backwards pass of nonlinear operations to incorporate a reference. Identifying which operations are nonlinear requires looking up each computational layer against an internal dictionary. Implementations will silently fail when encountering nonlinear operations outside the dictionary or when operations, usually activations, are re-used. Documentations or warnings are often absent. Users may only notice when the convergence deltas, which theoretically should be zero (but near machine precision in practice), become much higher, indicating that the attributions are incorrect. By contrast, tangermeme will never silently fail; it will raise a warning when convergence deltas are too high, can return the convergence deltas for monitoring and one can include custom nonlinear functions to avoid this issue altogether (Fig. 1d). Further documentation on this issue and how to resolve it is available alongside the rest of tangermeme’s documentation.

**Fig. 2:**
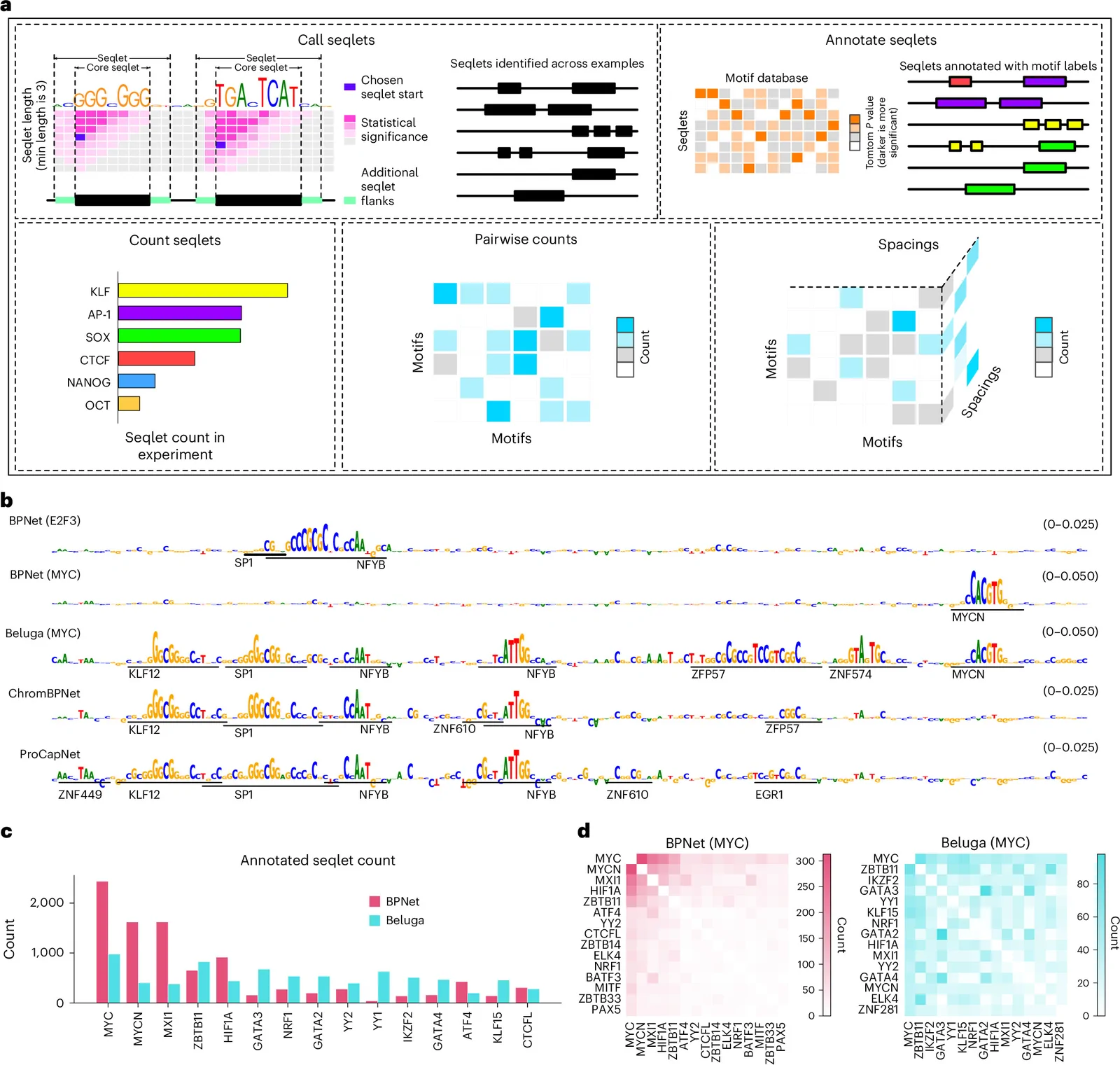
An automatic pipeline for the identification and use of seqlets. **a**, An overview of the implemented steps, beginning with the novel seqlet-calling algorithm, annotation of those seqlets using a motif database, annotation counting for each motif, counting the number of pairs of each annotation and, finally, considering spacings. **b**, Example attributions from several models at the PLD6 promoter along with seqlet calls (the bars underneath) and annotations. **c**, Motif counts for BPNet and Beluga models predicting MYC binding. **d**, Pairwise motif counts for the models in **c** showing the top 15 motifs for each.

In addition to implementing commonly used algorithms, tangermeme includes novel algorithms for automatically distilling learned *cis-*regulatory logic from trained deep learning models. These algorithms focus on the calling and use of ‘seqlets’, which are contiguous spans of high-attribution characters from an algorithm such as DeepLIFT/SHAP, in silico saturation mutagenesis, PISA^41^ or saliency^42^ (Fig. 2a). This seqlet caller calls variable-length seqlets directly, and it does so using a principled definition of a seqlet to provide a simple and robust algorithm (see Methods for details). After seqlets are called, tangermeme includes steps for annotating them using a motif database and then deriving statistics from these annotations, such as counts, pairwise presence and spacing relationships.

**Extended Data Fig. 1:**
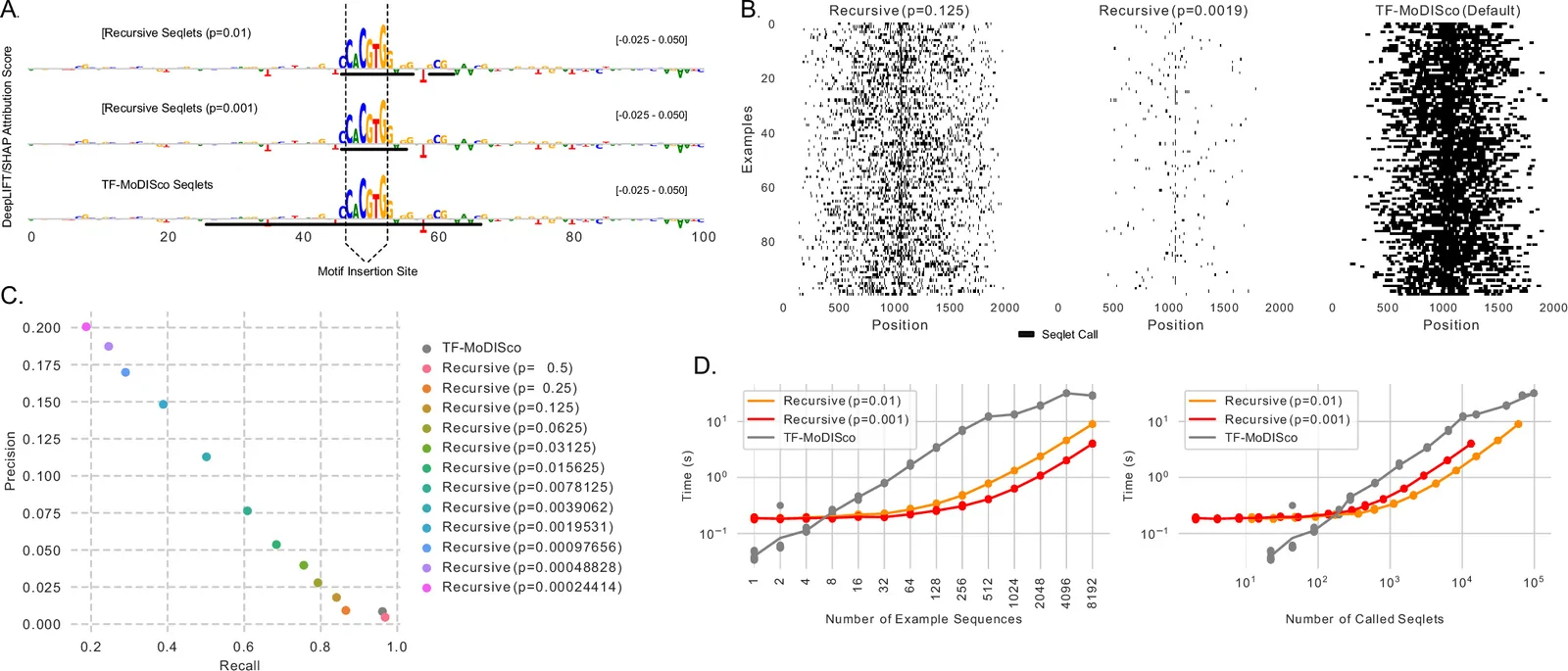
Comparison of the recursive and TF-MoDISco seqlet callers. (**A**) Seqlet calls using the recursive and TF-MoDISco seqlet callers on attributions for an example sequence where a MYC motif has been inserted into the center. (**B**) Spans for all seqlets called at two thresholds for the recursive seqlet caller, and for the TF-MoDISco seqlet caller, across 100 examples similar to A. (**C**) Precision and recall values measuring whether the called seqlet boundaries identify the MYC motif inserted into the middle of the sequence and only that, across a range of p-value threshlds for the recursive seqlet caller and also the TF-MoDISco seqlet caller. (**D**) Timings for the recursive and TF-MoDISco seqlet callers when run 10 times per step, shown as a function of the number of examples analyzed and by the number of seqlets ultimately called.

To better understand the properties of the seqlets identified by our approach, we compared them with those produced by a re-implementation of TF-MoDISco’s seqlet-calling agorithm (which is also included in tangermeme). As a simple synthetic dataset, we randomly generated 100 sequences, inserted the MYC motif into the center and used DeepLIFT/SHAP to generated attributions from a MYC BPNet model using the default settings. We observed that the seqlets called using the recursive algorithm were shorter than those called using TF-MoDISco’s approach, which have a long fixed default length (Extended Data Fig. 1a). Although both algorithms called more seqlets than just one in the middle, TF-MoDISco covered much more of the entire example with seqlet calls than even a very permission *P* value threshold (Extended Data Fig. 1b). As a more quantitative task, we then considered a binary classification task where the six base pairs (bp) of the inserted sequence were positive labels and the predictions were the binary spans of seqlet calls. Interestingly, when we considered a range of *P* value thresholds on this task, we observed a trend of precision and recall scores that TF-MoDISco falls near (Extended Data Fig. 1c), suggesting underlying similarities in the approaches. We then timed the two algorithms on calling seqlets on an increasing number of sequences and observed that, while TF-MoDISco is faster with a very small number of sequences, the recursive algorithm was faster for a modest-to-large number of sequences, both in terms of examples per second and seqlets per second (Extended Data Fig. 1d). An important note is that many of the design choices for the TF-MoDISco seqlet caller were made because it was part of a larger algorithm: TF-MoDISco calls wide seqlets because it can split them in subsequent steps and overcalls them because they can be easily discarded in the quality control steps. By contrast, the recursive seqlet caller is meant to be a stand-alone user-facing algorithm.

As a demonstration of these seqlet-based analysis capabilities, we used tangermeme to compare the learned logics of several models (see Methods for details). Attributions revealed that alternate sets of motifs drove predictions across different models at the promoter of PLD6 with, interestingly, different motifs driving MYC predictions from BPNet^9^ and Beluga^24^ (Fig. 2b). By automatically calling and annotating seqlets using these new algorithms, we can see that BPNet’s predictions are solely driven by a MYC motif, whereas Beluga is driven by many motifs that also drive accessibility and transcription initiation. We can then more comprehensively describe the motifs learned by these models by counting the annotated seqlets across all MYC peaks (Fig. 2c) and also through pairwise occurrences of these motifs (Fig. 2d). Through this automatic procedure, we can immediately see that the learned logics for BPNet and Beluga are significantly different despite being ostensibly trained to predict the same phenomena, with BPNet focusing almost exclusively on MYC motifs and Beluga learning a broader lexicon of motifs and interactions also associated with chromatin accessibility.

Tangermeme is a software package for genomic deep learning that offers efficient implementations of core algorithms as well as novel algorithms for distilling learned *cis-*regulatory patterns into insights. Its operations are easy to use, well documented and can be adapted or extended to research code in a variety of settings. Here, we have focused on how tangermeme can be used with models of TF binding and chromatin accessibility, but tangermeme can be used with models that make predictions for any modality, including alternative splicing, transcription, enhancer activity and even modalities for molecules other than DNA, such as RNA stability and binding.

We anticipate that well-documented, robust software packages such as tangermeme will be increasingly valuable as large language model-based coding agents become more common. When such systems can learn from examples of common genomic analyses and reuse tested, composable building blocks instead of recreating each analysis from scratch, there is less code for the researcher to audit. Accordingly, future development of tangermeme will focus not only on expanding its functionality but also on improving the documentation to provide comprehensive instructions for these systems on how to use the package without requiring the user to specify low-level application programming interface calls. Such integration will enable researchers to iterate quickly while still trusting the generated code.

## Methods

### One-hot encodings

Tangermeme introduces an optimized implementation of one-hot encoding a string of characters. In brief, this approach works by avoiding unnecessary intermediate calculation and, importantly, memory allocation, which can be costly when considering long or many strings. Specifically, when encountering a string of characters, many implementations first convert the characters into numeric indices before using these indices to set the location of the ones in the final one-hot encoded matrix, requiring the allocation of an intermediary array and a two-step procedure. In our approach, we avoid this intermediary step and interpret the byte representation of the character itself as an index that allows immediate one-hot encoding without the need for an intermediate array. Tangermeme also creates a one-hot encoded representation using unsigned 8-bit integers, which is significantly faster than encoding with signed 32- or 64-bit floats. Importantly, this approach will fail gracefully when encountering unexpected characters and still permits character sets to be ignored (usually *N*s) if desired.

We compared the speed of our one-hot encoding approach (in tangermeme v0.5.1) on the entirety of hg38 chr1 against several software packages. These included SeqPro (v0.6.1), CREsted (v1.4.0), gReLU (v1.0.7) and Selene SDK (0.6.0). We also used the one-hot encoding function in ChromBPNet (v1.0.1), although we did not install the entire package owing to its TensorFlow dependency.

Each package was timed solely on its respective one-hot encoding function. For several packages, including SeqPro and Selene SDK, there were steps involved with defining an alphabet that were not included in the timings. Each timing was performed on a single thread to avoid issues related to parallel processing that may have arisen.

### Models

In this work, we used five deep learning models, which are publicly available^43^. We trained the two BPNet models and are rehosting the other three models for convenience.

We trained BPNet models to predict the binding of E2F3 and MYC using the default parameters in the bpnet-lite v0.9.0 command-line tool. BPNet models make predictions for base-pair resolution profiles, which are the probability of an observed read mapping to each nucleotide in the output window of the model, and also for the log of the total number of read counts within the window. Respectively, these are the shape and the strength of the binding. These models were trained on chromatin immunoprecipitation followed by sequencing (ChIP-seq) experiments hosted on the ENCODE Compendium^44^ (ENCSR036QIR and ENCSR000EGJ for E2F3 and MYC, respectively, both using the control track ENCSR000BLJ). In each case, loci for training included those in the peaks file associated with each experiment along with GC-matched negatives computed from those peaks files. During training, 2,114-bp windows were extracted as input to the models that were centered on the peaks or chosen background regions with or without a randomly chosen jitter between −128 and 128. Half of the seen examples were reverse-complemented, meaning that both the nucleotide sequence and the base-pair resolution profiles were flipped along both the length and feature axes. A fixed count loss weight of 100 was used.

The DeepSEA/Beluga model was downloaded from Kipoi^45^ and modified to change the expected AGCT alphabet to be the more popular ACGT alphabet (http://kipoi.org/models/DeepSEA/beluga). We considered only the output for MYC in K562 (target 614, 0-indexed).

The ChromBPNet and ProCapNet models were downloaded from the ENCODE Compendium (https://www.encodeproject.org/annotations/ENCSR467RSV and https://www.encodeproject.org/annotations/ENCSR740IPL, respectively). In both cases, we used models from only fold 0.

### Simulations

In Fig. 1, we showed how tangermeme decouples sequence manipulations from the subsequent model operations to enable a richer space of analyses. In these simulations, we show predictions and attributions from the profile prediction output of a ChromBPNet model. For the marginalizations and ablations, we used the same set of 100 randomly generated DNA sequences that were 2,114-bp long using nucleotide probabilities derived from the genome (28% A/T, 22% C/G). For the marginalizations, we performed the respective operation with ChromBPNet before and after substituting ‘ATGATGTCAT’ into the middle of each of these sequences and display both, averaged across the sequences. For the ablations, we began by substituting that same subsequence into the sequences to generate the ‘before’ sequences and then dinucleotide shuffled the window between 1,030 bp and 1,075 bp to get the ‘after’ sequences. When running DeepLIFT/SHAP we used only one background shuffle per sequence because we are already averaging across the 100 randomly generated sequences. When running saturation mutagenesis, we perform a windowed version that focuses solely on the 1,040–1,075-bp window for speed without losing exactness, which surrounds where the motif is being substituted into. For the variant effect predictions, we change the nucleotide at position 1,053 from a G to an A.

We performed simulations to compare the seqlet caller from TF-MoDISco with the recursive seqlet caller. In these simulations, we randomly generated 100 sequences of length 2,114 with uniform nucleotide probabilities and substituted the motif ‘CACGTG’ into the center. We then used the MYC BPNet model to generate feature attribution scores for each using DeepLIFT/SHAP. This was implemented as marginalizing the MYC motif and using DeepLIFT/SHAP instead of prediction for the model operation step. We first ran the recursive seqlet caller with *P* value thresholds of 0.01 and 0.001, and the TF-MoDISco algorithm with default settings, on the set of 100 sequences and plotted a representative example (Extended Data Fig. 1a). We then reran the recursive seqlet caller with a range of *P* values ranging from 0.5 to 0.5^12^ and showed seqlet boundaries across all 100 examples for the *P* value threshold closest to 0.001, and a very permissive one at 0.125, and compared those with the default TF-MoDISco parameters. We then calculated precision and recall scores for each threshold by taking these masks that denoted seqlet boundaries and comparing them with labels that were set to 1 only for the central 6 bp, where the MYC motif was inserted. This comparison is imperfect in the sense that the seqlet callers would be correct if, by chance, MYC motifs occurred elsewhere in the sequence and drove model predictions in such a way that seqlets were called. However, this imperfection would penalize all seqlet-calling algorithms and does not prevent us from identifying broader trends.

Next, we timed the two algorithms when applied to a realistic seqlet-calling scenario. Specifically, we calculated attributions using DeepLIFT/SHAP and the same ChromBPNet model as before on K562 ATAC-seq peaks and then used the algorithms to call seqlets. We applied the algorithms to an increasing number of examples and timed them ten times at each step.

### Seqlet calling

Tangermeme includes a novel seqlet-calling algorithm that uses a recursive application of a basic statistical test to call seqlets from attribution scores. This recursive application makes it the first seqlet-calling algorithm to be able to call variable-length seqlets directly. In brief, a seqlet is a contiguous span of nucleotides with high attribution values. Our seqlet caller further defines a seqlet as ‘a span with a statistically significant attribution sum compared to a null distribution’, where all subspans within the seqlet would also be called seqlets according to this statistical test. This property does not need to hold for subspans below a certain length (by default 4) to prevent noise in the attributions from disrupting a seqlet call and to intentionally allow seqlets with uninformative characters within them.

This simple definition of a seqlet leads to a principled calling algorithm with only a few parameters: the *P* value threshold, the minimum and maximum seqlet lengths, optional additional flanks to add to the edges for robustness in downstream processing and the number of bins to use when converting the contiguous signal to a discrete one. Of these, the *P* value threshold is probably the most important to tune, although the minimum seqlet length may also matter depending on how noisy the attributions are. Changes to the maximum seqlet length do not affect seqlets that are smaller than the current maximum, and the number of bins is broadly robust across a range of values and mostly influences computational time and memory.

Conceptually, the algorithm proceeds as follows. First, the attributions are binned into a user-defined number of values and the frequency of attributions falling within each bin are calculated. These frequency histograms are then used to calculate null distributions for seqlets of each potential length. These null distributions encode the probability of observing binned attribution sums of each strength by chance, assuming that attribution values are independently observed and identically distributed across positions. These nulls can then be used to directly compute *P* values by summing the probability density more extreme than the observed attribution sums across the candidate seqlets. By comprehensively considering all starting positions and all seqlet lengths in a user-defined range, we can then decode the *P* values to identify the longest non-overlapping seqlets that meet our criteria.

More formally, the algorithm proceeds as follows. We begin with attribution values $X\in {{\mathbb{R}}}^{n,l}$ where *n* is the number of examples and *l* is the length of these examples. In principle, *l* does not need to be constant across examples but in our current implementation it is assumed to be fixed. These attribution values are then binned by min-max scaling them using the global minimum and maximum values, multiplying by the number of bins *b*, and taking the floor of the resulting value.

1 $${\alpha }_{i,\;j}=\left\lfloor (b-1)\,\frac{{X}_{i,\;j}-\mathop{\min }\limits_{i,\;j}X}{\mathop{\max }\limits_{i,\;j}X-\mathop{\min }\limits_{i,\;j}X}\right\rfloor .$$

These integerized scores are then used to create a frequency histogram $f\in {{\mathbb{R}}}^{b}$:

2 $${f}_{i}=\frac{1}{nl}\sum _{j=1}^{n}\sum _{k=1}^{l}\delta ({\alpha }_{j,k}=i),$$

where *δ* is the Kronecker delta function, which is 1 when the term is true and 0 otherwise. This is similar to the initial steps in the procedure in Tomtom^46^ for calculating null distributions.

This frequency histogram is then used to calculate null distributions for each seqlet length. These null distributions essentially answer the question ‘what attribution sum would I get by randomly choosing this number of (integerized versions of the) attribution values from the data?’ without considering whether the values are contiguous. Each null distribution encodes a probability density function (PDF) over integerized scores, with the span of these scores increasing with length. Specifically, each null distribution spans between 0 and *tb* with *t* being the seqlet length. For simplicity, we will represent all of these PDFs in a single sparse matrix $A\in {{\mathbb{R}}}^{T,Tb}$ where *T* is the maximum seqlet length and *A*_1,0:*b*_ is set to be equal to *f* and the rest of *A* is filled in by

3 $${A}_{\!j,k}=\sum _{l=1}^{t}{A}_{\!j-1,k-l}{\;f}_{l}.$$

Although this procedure is iterative in nature, it is not the inspiration for the use of ‘recursive’ in the name of the seqlet caller.

Each of these null distributions is currently a PDF. As we will need to quickly calculate *P* values, we convert these PDFs into 1 − CDFs (where CDF is cumulative distribution function), from which determining *P* values is a simple indexing operation.

4 $${B}_{\!j,k}=1-\sum _{i=1}^{k}{A}_{\!j,i}.$$

Given these 1 − CDFs, we can then convert our original attributions *X* into *P* values by binning them using the same procedure as before and indexing into these *B* distributions. This yields $P\in {{\mathbb{R}}}^{n,l}$ comprising *P* values for each position and each potential seqlet span length.

Seqlets are then called from *P* on the basis of the definition that seqlets are entries in *P* whose value falls below the threshold and also where all subspans (longer than the minimum seqlet span) within the seqlet also fall below the threshold. For efficiency, this is done by setting the first rows between 0 and min_seqlet_len to 0 and then setting each entry *P*_*i*,*j*_ to ${P}_{i,\;j}={\mathop{\max }\nolimits_{i}}\;{P}_{i,\;j}$. Now, by indexing into each element, we can know whether all spans beginning at that position pass the *P* value threshold. We can do this efficiently by proceeding from *i* = 0 to *i* = max_seqlet_len and setting ${P}_{i,\;j}=\max ({P}_{i,\;j},{P}_{i-1,\;j})$. If any spans are not statistically significant, this procedure will block any longer spans from being considered. To consider all subspans and not just those starting at position *j*, we then proceed diagonally from right to left, setting ${P}_{i,\;j}=\max ({P}_{i,\;j},{P}_{i-i,\;j+1})$. Together, these two steps mean that when the value at *P*_*i*,*j*_ is below the threshold, all subspans within the width would be called as statistically significant. Accordingly, we can proceed to call seqlets by iterating from the maximum seqlet length to the minimum seqlet length, checking whether the minimum *P* value for that row is below the threshold and, when identifying a seqlet, setting all columns within the width of the seqlet to 1 to prevent multiple seqlets from being called in the same position. This procedure means that the longest seqlet will be called for a given set of positions, not necessarily the most statistically significant.

### Seqlet annotation

Once seqlets have been called using the recursive seqlet caller, they are then annotated with the motif they most resemble. This is done using the annotate_seqlets command. This annotation procedure involves using Tomtom-lite^47^ to map the nucleotide sequence within the seqlet to a provided motif database. In these evaluations, the motif database was the slice of the JASPAR2024^48^ database derived from ChIP-seq experiments performed in *Homo sapiens*. Seqlets were annotated with the motif with the lowest *P* value.

### Seqlet counting

After seqlets have been called and annotated, basic statistics can be extracted from the presence of these annotated seqlets across sequences. We can count the number of times each annotation appears in each sequence to get a $C\in {{\mathbb{Z}}}^{n,m}$ matrix, where *n* is the number of sequences and *m* is the number of motifs in the motif database. This matrix can then be summed across the first dimension to count the number of times each motif is present or across the second dimension to get the number of seqlets called per sequence. We show the first of these statistics for the BPNet (MYC) and Beluga (MYC) models in Fig. 2c.

Going further, we can also count the number of times each pair of motifs appear within the same sequence and get $P\in {{\mathbb{Z}}}^{n,m,m}$. There are several modes this procedure can be run in. First, it can be run asymmetrically or symmetrically. When run symmetrically, the presence of motifs A and B is counted in the row associated with A and also in the row associated with B, yielding a matrix that is symmetric. When run asymmetrically, motifs are counted according to their order in the sequence from the start to the end, not accounting for the strandedness of the motif. This can be useful when the ordering of motifs is important, such as for transcription initiation, although one must manually ensure that the strandedness is correct. The default is running symmetrically. The second mode involves whether motif hits are unique or not; for example, when the motifs within a sequence are ‘A A A B’, whether we record three ‘A B’ pairs or just a single one. This can be particularly important when dealing with arrays of motifs, such as KLF, because the count of those array elements may end up being significantly higher than others.

Finally, we can also consider the spacing between motifs. Here, we create a count of each pair of motifs at each possible spacing up until a user-defined maximum. All spacings after that maximum are ignored. Similar to the pairwise motif counts, this matrix can be symmetric or not. This matrix will probably be extremely sparse, as many motifs do not co-occur at all, and even those that do co-occur are unlikely to do so frequently enough to fill in many potential spacings.

### Reporting summary

Further information on research design is available in the Nature Portfolio Reporting Summary linked to this article.

## Online content

Any methods, additional references, Nature Portfolio reporting summaries, source data, extended data, supplementary information, acknowledgements, peer review information; details of author contributions and competing interests; and statements of data and code availability are available at https://doi.org/10.1038/s41592-026-03254-z.

## Supplementary information

Reporting Summary

Peer Review File

## Data Availability

For our large-scale seqlet-calling analysis, we used peak calls from a publicly available ChIP-seq experiment performed on MYC binding in K562, available on the ENCODE Portal (ENCFF114VAI).

## Code availability

Tangermeme is a free and open source software available under the MIT license at https://github.com/jmschrei/tangermeme. The latest version can be installed via pip using pip install tangermeme. Documentation and tutorials are available at https://tangermeme.readthedocs.io/en/latest/, including tutorials that produce Figs. 1 and 2 and Extended Data Fig. 1.

## References

[1] Winters-Hilt, S. Hidden markov model variants and their application. *BMC Bioinformatics*, **7****Suppl 2(S2)**, S14 (2006). 10.1186/1471-2105-7-S2-S14 PMC1683574 17118135

[2] Huang, S. et al. Applications of support vector machine (SVM) learning in cancer genomics. *Cancer Genomics Proteomics* **15**, 41–51 (2018). 10.21873/cgp.20063 PMC5822181 29275361

[3] Noble, W. S. What is a support vector machine?. *Nat. Biotechnol.* **24**, 1565–1567 (2006). 10.1038/nbt1206-1565 17160063

[4] Libbrecht, M. W. & Noble, W. S. Machine learning applications in genetics and genomics. *Nat. Rev. Genet.* **16**, 321–332 (2015). 10.1038/nrg3920 PMC5204302 25948244

[5] Bepler, T. & Berger, B. Learning the protein language: evolution, structure, and function. *Cell Syst.* **12**, 654–669 (2021). 10.1016/j.cels.2021.05.017 PMC8238390 34139171

[6] Velten, B. & Stegle, O. Principles and challenges of modeling temporal and spatial omics data. *Nat. Methods* **20**, 1462–1474 (2023). 10.1038/s41592-023-01992-y 37710019

[7] Eraslan, G., Avsec, Ž, Gagneur, J. & Theis, F. J. Deep learning: new computational modelling techniques for genomics. *Nat. Rev. Genet.* **20**, 389–403 (2019). 10.1038/s41576-019-0122-6 30971806

[8] Hu, X., Fernie, A. R. & Yan, J. Deep learning in regulatory genomics: from identification to design. *Curr. Opin. Biotechnol.* **79**, 102887 (2023). 10.1016/j.copbio.2022.102887 36640453

[9] Avsec, Ž et al. Base-resolution models of transcription-factor binding reveal soft motif syntax. *Nat. Genet.* **53**, 354–366 (2021). 10.1038/s41588-021-00782-6 PMC8812996 33603233

[10] Yin, Q., Wu, M., Liu, Q., Lv, H. & Jiang, R. DeepHistone: a deep learning approach to predicting histone modifications. *BMC Genomics* **20**, 193 (2019). 10.1186/s12864-019-5489-4 PMC6456942 30967126

[11] Kelley, D. R., Snoek, J. & Rinn, J. L. Basset: learning the regulatory code of the accessible genome with deep convolutional neural networks. *Genome Res.* **26**, 990–999 (2016). 10.1101/gr.200535.115 PMC4937568 27197224

[12] Pampari, A. et al. ChromBPNet: bias factorized, base-resolution deep learning models of chromatin accessibility reveal *cis*-regulatory sequence syntax, transcription factor footprints and regulatory variants. Preprint at *bioRxiv* 10.1101/2024.12.25.630221 (2025).

[13] Hu, Y. et al. Multiscale footprints reveal the organization of cis-regulatory elements. *Nature* **638**, 779–786 (2025). 10.1038/s41586-024-08443-4 PMC11839466 39843737

[14] Zhou, J. Sequence-based modeling of three-dimensional genome architecture from kilobase to chromosome scale. *Nat. Genet.* **54**, 725–734 (2022). 10.1038/s41588-022-01065-4 PMC9186125 35551308

[15] Fudenberg, G., Kelley, D. R. & Pollard, K. S. Predicting 3D genome folding from DNA sequence with akita. *Nat. Methods* **17**, 1111–1117 (2020). 10.1038/s41592-020-0958-x PMC8211359 33046897

[16] De Almeida, B. P., Reiter, F., Pagani, M. & Stark, A. DeepSTARR predicts enhancer activity from DNA sequence and enables the de novo design of synthetic enhancers. *Nat. Genet.* **54**, 613–624 (2022). 10.1038/s41588-022-01048-5 35551305

[17] Cochran, K. et al. Dissecting the *cis*-regulatory syntax of transcription initiation with deep learning. Preprint at *bioRxiv* 10.1101/2024.05.28.596138 (2024).

[18] Jaganathan, K. et al. Predicting splicing from primary sequence with deep learning. *Cell* **176**, 535–548 (2019). 10.1016/j.cell.2018.12.015 30661751

[19] Zeng, T. & Li, Y. I. Predicting RNA splicing from DNA sequence using pangolin. *Genome Biol.* **23**, 103 (2022). 10.1186/s13059-022-02664-4 PMC9022248 35449021

[20] Kanuparthi, B. et al. Sequence based prediction of cell type specific microRNA binding and mRNA degradation for therapeutic discovery. Preprint at *bioRxiv* 10.1101/2025.05.15.654105 (2025).

[21] Agarwal, V. & Kelley, D. R. The genetic and biochemical determinants of mRNA degradation rates in mammals. *Genome Biol.* **23**, 245 (2022). 10.1186/s13059-022-02811-x PMC9684954 36419176

[22] Novakovsky, G., Dexter, N., Libbrecht, M. W., Wasserman, W. W. & Mostafavi, S. Obtaining genetics insights from deep learning via explainable artificial intelligence. *Nat. Rev. Genet.* **24**, 125–137 (2023). 10.1038/s41576-022-00532-2 36192604

[23] Wang, X. et al. Deep learning approaches for non-coding genetic variant effect prediction: current progress and future prospects. *Brief. Bioinform.* **25**, bbae446 (2024). 10.1093/bib/bbae446 PMC11401448 39276327

[24] Zhou, J. & Troyanskaya, O. G. Predicting effects of noncoding variants with deep learning-based sequence model. *Nat. Methods* **12**, 931–934 (2015). 10.1038/nmeth.3547 PMC4768299 26301843

[25] Muiños, F., Martínez-Jiménez, F., Pich, O., Gonzalez-Perez, A. & Lopez-Bigas, N. In silico saturation mutagenesis of cancer genes. *Nature* **596**, 428–432 (2021). 10.1038/s41586-021-03771-1 34321661

[26] Shrikumar, A., Greenside, P. & Kundaje, A. Learning important features through propagating activation differences. Preprint at 10.48550/arXiv.1704.02685 (2017).

[27] Koo, P. K., Majdandzic, A., Ploenzke, M., Anand, P. & Paul, S. B. Global importance analysis: an interpretability method to quantify importance of genomic features in deep neural networks. *PLoS Comput. Biol.* **17**, e1008925 (2021). 10.1371/journal.pcbi.1008925 PMC8118286 33983921

[28] Toneyan, S. & Koo, P. K. Interpreting *cis*-regulatory interactions from large-scale deep neural networks. *Nat Genet.* **56**, 2517–2527 (2024). 10.1038/s41588-024-01923-3 PMC12065635 39284975

[29] Schreiber, J. et al. Programmatic design and editing of *cis*-regulatory elements. Preprint at *bioRxiv* 10.1101/2025.04.22.650035 (2025).

[30] Linder, J. & Seelig, G. Fast activation maximization for molecular sequence design. *BMC Bioinformatics* **22**, 510 (2021). 10.1186/s12859-021-04437-5 PMC8527647 34670493

[31] De Winter, S., Konstantakos, V. & Aerts, S. Modelling and design of transcriptional enhancers. *Nat. Rev. Bioeng.* **3**, 374–389 (2025).

[32] Kokhlikyan, N. et al. Captum: a unified and generic model interpretability library for PyTorch. Preprint at 10.48550/arXiv.2009.07896 (2020).

[33] Chen, K. M., Cofer, E. M., Zhou, J. & Troyanskaya, O. G. Selene: a PyTorch-based deep learning library for sequence data. *Nat. Methods* **16**, 315–318 (2019). 10.1038/s41592-019-0360-8 PMC7148117 30923381

[34] Avsec, Z. et al. The Kipoi repository accelerates community exchange and reuse of predictive models for genomics. *Nat. Biotechnol.* **37**, 592–600 (2019). 10.1038/s41587-019-0140-0 PMC6777348 31138913

[35] Kempynck, N. et al. CREsted: modeling genomic and synthetic cell-type-specific enhancers across tissues and species. *Nat. Methods* **23**, 946–959 10.1038/s41592-026-03057-2 (2026). PMC13167471 41927920

[36] Klie, A. et al. Predictive analyses of regulatory sequences with EUGENe. *Nat. Comput. Sci.* **3**, 946–956 (2023). 10.1038/s43588-023-00544-w PMC10768637 38177592

[37] Lal, A., Gunsalus, L., Nair, S., Biancalani, T. & Eraslan, G. gReLU: a comprehensive framework for DNA sequence modeling and design. *Nat. Methods* **22**, 2253–2257 10.1038/s41592-025-02868-z (2025). PMC12615257 41094274

[38] Shrikumar, A. et al. Technical note on transcription factor motif discovery from importance scores (TF-MoDISco) version 0.5.6.5. Preprint at 10.48550/arXiv.1811.00416 (2018).

[39] Harris, C. R. et al. Array programming with NumPy. *Nature* **585**, 357–362 (2020). 10.1038/s41586-020-2649-2 PMC7759461 32939066

[40] Pedregosa, F. et al. Scikit-learn: machine learning in Python. *J. Mach. Learn. Res*. **12**, 2825–2830 (2011).

[41] McAnany, C. E. et al. Positional interpretation of cis-regulatory code and nucleosome organization with deep learning models. *Nat. Commun.* **17**, 8326 10.1038/s41467-026-74807-1 (2026). PMC13469612 42401553

[42] Majdandzic, A., Rajesh, C. & Koo, P. K. Correcting gradient-based interpretations of deep neural networks for genomics. *Genome Biol.* **24**, 109 (2023). 10.1186/s13059-023-02956-3 PMC10169356 37161475

[43] Schreiber, J. Genomic deep learning models used in tangermeme paper. *Zenodo*10.5281/zenodo.16751681 (2025).

[44] ENCODE Project Consortium et al. Expanded encyclopaedias of DNA elements in the human and mouse genomes. *Nature* **583**, 699–710 (2020). 10.1038/s41586-020-2493-4 PMC7410828 32728249

[45] Avsec, Ž et al. The Kipoi repository accelerates community exchange and reuse of predictive models for genomics. *Nat. Biotechnol.* **37**, 592–600 (2019). 10.1038/s41587-019-0140-0 PMC6777348 31138913

[46] Gupta, S., Stamatoyannopoulos, J. A., Bailey, T. L. & Noble, W. S. Quantifying similarity between motifs. *Genome Biol.* **8**, R24 (2007). 10.1186/gb-2007-8-2-r24 PMC1852410 17324271

[47] Schreiber, J. Tomtom-lite: accelerating Tomtom enables large-scale and real-time motif similarity scoring. *Bioinformatics* **41**, btaf577 10.1093/bioinformatics/btaf577 (2025). PMC12619998 41124023

[48] Rauluseviciute, I. et al. JASPAR 2024: 20th anniversary of the open-access database of transcription factor binding profiles. *Nucleic Acids Res.* **52**, D174–D182 (2024). 10.1093/nar/gkad1059 PMC10767809 37962376

