# Supplementary material for: Tangermeme: a toolkit for understanding cis-regulatory logic using deep learning models
Source: Nat Methods. 2026 Oct 8;23(11):2136–40. doi: 10.1038/s41592-026-03254-z (PMC13736675; doi:10.1038/s41592-026-03254-z)
Supplement: Supplementary file 2 [file 41592_2026_3254_MOESM2_ESM.pdf]

nature portfolio

Peer Review File

tangermeme: A toolkit for understanding cis-regulatory logic using deep learning models

Corresponding Author: Dr Jacob Schreiber

Version 0:

Decision Letter:

17th Oct 2025

Dear Dr Schreiber,

Your Brief Communication, "tangermeme: A toolkit for understanding cis-regulatory logic using deep learning models", has now been seen by 3 reviewers. As you will see from their comments below, although the reviewers find your work of considerable potential interest, they have raised a number of concerns. We continue to be interested in potentially publishing your manuscript in Nature Methods, but would like to evaluate your response to these concerns before we reach a final decision on publication.

We therefore invite you to revise your manuscript to fully address all these concerns.

We are committed to providing a fair and constructive peer-review process. Do not hesitate to contact us if there are specific requests from the reviewers that you believe are technically impossible or unlikely to yield a meaningful outcome.

When revising your paper:

* include a point-by-point response to the reviewers and to any editorial suggestions

* please underline/highlight any additions to the text or areas with other significant changes to facilitate review of the revised manuscript

* address the points listed described below to conform to our open science requirements

* ensure it complies with our general format requirements as set out in our guide to authors at www.nature.com/naturemethods

* resubmit all the necessary files by using the link below to access your home page

Link Redacted

Note: This URL links to your confidential account and associated information about manuscripts you may have submitted, or that you are reviewing for us. If you wish to forward this email to co-authors, please delete this link.

We hope to receive your revised paper within 3 months. If you are substantially delayed, please let us know. In this event, we will still be happy to reconsider your paper at a later date so long as nothing similar has been accepted for publication at Nature Methods or published elsewhere.

OPEN SCIENCE REQUIREMENTS

REPORTING SUMMARY
When revising your manuscript, please update your reporting summary.

Reporting summary: https://www.nature.com/documents/nr-reporting-summary.zip

If your paper includes custom software, we also ask you to complete a supplemental reporting summary.

Software supplement: https://www.nature.com/documents/nr-software-policy.pdf

Please submit these with your revised manuscript. They will be available to reviewers to aid in their evaluation if the paper is re-reviewed. If you have any questions about the checklist, please see http://www.nature.com/authors/policies/availability.html or contact me.

Please note that these forms are dynamic 'smart pdfs' and must therefore be downloaded and completed in Adobe Reader. We will then flatten them for ease of use by the reviewers. If you would like to reference the guidance text as you complete the template, please access these flattened versions at http://www.nature.com/authors/policies/availability.html.

For any revision that includes light microscopy data, we ask our authors to please include a completed light microscopy reporting table [https://www.nature.com/documents/Light_microscopy_reporting_table.xlsx] to ensure the methods are described thoroughly. The table will be available to reviewers and ultimately published should the manuscript be accepted at the journal.

EXTENDED DATA FIGURES
When re-submitting your manuscript, please ensure that any supplementary figures and tables that are crucial to the manuscript's conclusions are converted into Extended Data figures and tables to increase visibility of these data. Extended Data figures and tables are online-only (present in the online PDF and full-text HTML versions of the paper), peer-reviewed display items that provide essential background to the article but are not included in the main article due to space constraints. A maximum of ten Extended Data display items (figures and tables) is permitted.

DATA AVAILABILITY
We strongly encourage you to deposit all new data associated with the paper in a persistent repository where they can be freely and enduringly accessed. We recommend submitting the data to discipline-specific and community-recognized repositories; a list of repositories is provided here: http://www.nature.com/sdata/policies/repositories

All novel DNA and RNA sequencing data, protein sequences, genetic polymorphisms, linked genotype and phenotype data, gene expression data, macromolecular structures, and proteomics data must be deposited in a publicly accessible database, and accession codes and associated hyperlinks must be provided in the "Data Availability" section.

For papers containing bioimaging data, we strongly recommend depositing the data to Bioimage Archive (https://www.ebi.ac.uk/bioimage-archive/). Associated accession codes and hyperlinks should be provided in the "Data Availability" section.

Refer to our data policies here: https://www.nature.com/nature-research/editorial-policies/reporting-standards#availability-of-data

To further increase transparency, we encourage you to provide, in tabular form, the data underlying the graphical representations used in your figures. This is in addition to our data-deposition policy for specific types of experiments and large datasets. For readers, the source data will be made accessible directly from the figure legend. Spreadsheets can be submitted in .xls, .xlsx or .csv formats. Only one (1) file per figure is permitted: thus if there is a multi-paneled figure the source data for each panel should be clearly labeled in the csv/Excel file; alternately the data for a figure can be included in multiple, clearly labeled sheets in an Excel file. File sizes of up to 30 MB are permitted. When submitting source data files with your manuscript please select the Source Data file type and use the Title field in the File Description tab to indicate which figure the source data pertains to.

Please include a "Data availability" subsection in the Online Methods. This section should inform readers about the availability of the data used to support the conclusions of your study, including accession codes to public repositories, references to source data that may be published alongside the paper, unique identifiers such as URLs to data repository entries, or data set DOIs, and any other statement about data availability. At a minimum, you should include the following statement: "The data that support the findings of this study are available from the corresponding author upon request", describing which data is available upon request and mentioning any restrictions on availability. If DOIs are provided, please include these in the Reference list (authors, title, publisher (repository name), identifier, year). For more guidance on how to write this section please see: http://www.nature.com/authors/policies/data/data-availability-statements-data-citations.pdf

CODE AVAILABILITY
Please include a "Code Availability" subsection in the Online Methods which details how your custom code is made available. Only in rare cases (where code is not central to the main conclusions of the paper) is the statement "available upon request" allowed (and reasons should be specified).

We request that you deposit code in a DOI-minting repository such as Zenodo, Gigantum or Code Ocean and cite the DOI in the Reference list. We also request that you use code versioning and provide a license.

For more information on our code sharing policy and requirements, please see:
https://www.nature.com/nature-research/editorial-policies/reporting-standards#availability-of-computer-code

MATERIALS AVAILABILITY
As a condition of publication in Nature Methods, authors are required to make unique materials promptly available to others without undue qualifications.

Authors reporting new chemical compounds must provide chemical structure, synthesis and characterization details. Authors reporting mutant strains and cell lines are strongly encouraged to use established public repositories.

More details about our materials availability policy can be found at https://www.nature.com/nature-portfolio/editorial-policies/reporting-standards#availability-of-materials

ORCID
Nature Methods is committed to improving transparency in authorship. As part of our efforts in this direction, we are now requesting that all authors identified as 'corresponding author' on published papers create and link their Open Researcher and Contributor Identifier (ORCID) with their account on the Manuscript Tracking System (MTS), prior to acceptance. This applies to primary research papers only. ORCID helps the scientific community achieve unambiguous attribution of all scholarly contributions. You can create and link your ORCID from the home page of the MTS by clicking on 'Modify my Springer Nature account'. For more information please visit please visit www.springernature.com/orcid.

Please do not hesitate to contact me if you have any questions or would like to discuss these revisions further. We look forward to seeing your revised manuscript and thank you for the opportunity to consider your work.

Best regards,

Lin Tang, PhD
Senior Editor
Nature Methods

Reviewers' Comments:

Reviewer #1 (Remarks to the Author):

This manuscript introduces tangermeme, a comprehensive Python toolkit for post-training analysis of genomic deep learning models. The author addresses an important gap in the field by providing optimized, model-agnostic implementations of interpretation methods alongside novel algorithms for seqlet calling and annotation.

Major Strengths
- The focus on "everything-but-the-model" is well-motivated. The author correctly identifies that while model training tools exist, comprehensive post-training analysis frameworks are lacking.

- The optimizations are impressive, one-hot encoding hg38 chr1 in under 2 seconds represents very useful and practical improvement. The built-in batching for DeepLIFT/SHAP removes memory constraints while maintaining exactness, addressing a real limitation in existing tools.

- The recursive seqlet caller is a genuine contribution, being the first to call variable-length seqlets using a principled statistical framework.

- The separation of sequence manipulations from model operations (Figure 1D) elegantly enables previously difficult analyses, such as computing attributions after variant substitution.

Areas for Improvement

- Overall, I believe that the manuscript would benefit from defining technical terms (e.g., marginalization, screening, convergence deltas etc) and providing brief contextual explanations to make it accessible to the broader Nature Methods readership beyond specialists in genomic deep learning. More importantly, explaining how exactly the mentioned operations serve the purpose of "distilling learned cis-regulatory patterns into insights" would improve the manuscript ie. why are these operations critical and how are they useful.

- Figure 1A's comparison table would benefit from brief definitions of each functionality category
The convergence delta comparison (Figure 1B) is compelling but needs more explanation of why other implementations fail and why this is a critical metric.

- I think Figure 1D is extremely confusing and can be improved. It is unclear why the same "before" sequence has different profiles. It is unclear why the "before" profile of ablation is same as the "after" profile of the marginalization. Maybe what is meant is that the "before" sequences in ablation and VEP contains the blue AP-1 motif so that the implanted AP-1 is ablated and substituted? Does the ablation sequence have a meaning? It would be very useful if the caption elaborates that is the goal of these operations and how they are done. More importantly, beyond "what these operations are" the audience should be informed about "why these operations matter".

- Mentioning Kipoi et al (kipoi-interpret, kipoi-seq, kipoi-veff etc) in Figure 1 would be fair since it is (or was) capable of some of these operations, although it is not maintained any more.

- To enhance the clarity of the figures, I suggest expanding the captions for both Figure 1 and Figure 2. Specifically, for Figure 2A, the high information density makes it challenging to interpret. The caption could be improved by explicitly defining the elements, such as clarifying what the rows in the 'seqlet calling panel' represent (for instance, are they iterations of recursion?). Additionally, if important, please provide a legend or description for the gray coloring used in the subsequent panels.

- Figure 2D Beluga panel can be improved by ordering rows and columns by clustering. If there is anything biologically interesting or well-known in the pairs, it would be good to highlight that.

- The seqlet calling algorithm (pages 7-8) is great but mathematically dense. An intuitive explanation and/or a pseudocode before the formalism would improve accessibility.

-The demonstrations focus on TF binding and chromatin accessibility. Also mentioning that other modalities (RNA stability) can benefit from this toolkit would better showcase the toolkit's generalizability.

Recommendation
This is solid methods development work that addresses a real need in genomic deep learning. The software appears well-engineered, documented, and benchmarked. With revisions improving its accessibility for the general audience, this would be a valuable contribution to Nature Methods. The toolkit has clear potential for broad community adoption.

Reviewer #2 (Remarks to the Author):

Summary of key results

This work introduces tangermeme, an open-source toolkit for analyzing and interpreting sequence-to-function DNA models. The software decouples sequence manipulation (e.g., scoring genetic variants or ablating motifs) from model operations (e.g., generating predictions or feature attributions). This modular design allows researchers to "stack" these operations, such as calculating how a specific variant alters the importance scores of nearby motifs. The toolkit provides a complete workflow to uncover learned cis-regulatory logic by identifying "seqlets" (contiguous, high-importance nucleotides) from attribution scores, annotating seqlets by mapping to known TF motifs, and performing various statistical analysis of motif occurrence patterns to reveal regulatory syntax.

Originality and significance

The novelty of this work is the creation of a generalized end-to-end package for the post-training analysis and interpretation of genomic deep learning models. While existing frameworks focus on model training (e.g., Selene, EUGENe) or implement a small subset of interpretation algorithms (e.g., Captum), tangermeme provides a comprehensive solution. Additionally, the author introduces a recursive variable-length seqlet calling method.

Interestingly, this work highlights that different models (e.g. BPNet vs. Beluga) predicting the same phenomena (e.g. MYC binding) can reveal different regulatory motif logic learned by the model. This is a valuable example highlighting that attributions computed using different models can lead to different conclusions about underlying biology.

This work is presented clearly and concisely, uses appropriate statistical tests, gives appropriate credit to previous work, and provides references to data, models, and scripts necessary for reproduction.

Suggested improvements and points to address:

The author states “These operations have built-in batching, support for alternative data types and devices, and work out-of-the-box on multi-input/output models” (see line 50). To improve clarity, could the authors elaborate on the practical limitations of this feature? For example, are there constraints on model architecture or block types? Could models with very long or variable-length inputs pose compatibility challenges? We suggest adding a brief note on tangermeme’s compatibility principles to

clarify the expected scope of supported sequence-function models.

Furthermore, the paper could further clarify its design philosophy for model access and integration. Is the toolkit designed for seamless integration with model repositories like Kipoi, or is the user generally responsible for loading any compatible model object into memory first?

The authors report that tangermeme's one-hot encoding is more efficient and almost 3x faster than the closest best algorithm (line 61). However, the corresponding "One-hot Encodings" subsection in the Methods only describes the benchmarking procedure, not the implementation itself. Given this significant performance gain, please add a brief description of the technical optimizations or algorithmic approach.

The authors introduce a novel algorithm to call variable-length seqlets directly from attribution scores. To validate this new method, the paper should include a quantitative benchmark against a common alternative, such as the workflow in TF-MoDISco. Key metrics to measure could include computational efficiency, identified seqlets' quality, and robustness to parameter choices.

The authors position tangermeme among existing toolkits like Selene and EUGENe, in a domain where wide software adoption has been challenging. What lessons from prior tools informed its design, and which key features or performance advantages are expected to overcome these adoption hurdles?

The Ledidi column in Figure 1a appears to indicate that it is incompatible with tangermeme. Is that correct?

Reviewer #2 (Remarks on code availability):

The software and code looks very well done.

Reviewer #3 (Remarks to the Author):

I co-reviewed this manuscript with one of the reviewers who provided the listed reports. This is part of the Nature Methods initiative to facilitate training in peer review and to provide appropriate recognition for Early Career Researchers who co-review manuscripts.

Reviewer #3 (Remarks on code availability):

The GitHub repository contains an easily installable package with examples, comprehensive documentation (README and Readthedocs), and exhaustive Jupyter Notebook tutorials covering every tool described in the manuscript. I was able to install and successfully run modules of the package.

Version 1:

Decision Letter:

Our ref: NMETH-BC62472A

8th May 2026

Dear Dr. Schreiber,

Thank you for submitting your revised manuscript "tangermeme: A toolkit for understanding cis-regulatory logic using deep learning models" (NMETH-BC62472A). We deeply apologize for the longer peer review process than we would hope. This paper has now been seen again by Reviewer 2. (Other reviewers haven't sent their review comments despite multiple reminders.) After evaluation and discussion within the editorial team, we'll be happy in principle to publish it in Nature Methods, pending minor revisions to comply with our editorial and formatting guidelines.

We are now performing detailed checks on your paper and will send you a checklist detailing our editorial and formatting requirements within two weeks or so. Please do not upload the final materials and make any revisions until you receive this additional information from us.

TRANSPARENT PEER REVIEW
Nature Methods offers a transparent peer review option for new original research manuscripts submitted from 17th February 2021. We encourage increased transparency in peer review by publishing the reviewer comments, author rebuttal letters and editorial decision letters if the authors agree. Such peer review material is made available as a supplementary peer review file. **Please state in the cover letter 'I wish to participate in transparent peer review' if you want to opt in, or 'I do not wish to participate in transparent peer review' if you don't.** Failure to state your preference will result in delays in accepting your manuscript for publication.

Please note: we allow redactions to authors' rebuttal and reviewer comments in the interest of confidentiality. If you are concerned about the release of confidential data, please let us know specifically what information you would like to have removed. Please note that we cannot incorporate redactions for any other reasons. Reviewer names will be published in the

peer review files if the reviewer signed the comments to authors, or if reviewers explicitly agree to release their name. For more information, please refer to our FAQ page.

ORCID
IMPORTANT: Non-corresponding authors do not have to link their ORCIDs but are encouraged to do so. Please note that it will not be possible to add/modify ORCIDs at proof. Thus, please let your co-authors know that if they wish to have their ORCID added to the paper they must follow the procedure described in the following link prior to acceptance:
https://www.springernature.com/gp/researchers/orcid/orcid-for-nature-research

Author names using non-Roman characters
Nature Portfolio journals can support presentation of author names using non-Roman characters in the HTML version of the article. If you wish to, please include author names in parentheses after the Roman-character spelling; see example online here. Currently supported scripts are: Arabic, Chinese, Cyrillic, Devanagari, Greek, Hebrew, Hangul, Japanese and Persian. You will be asked to verify the rendering is correct at proof stage.

Thank you again for your interest in Nature Methods. Please do not hesitate to contact me if you have any questions. We will be in touch again soon.

Sincerely,

Lin Tang, PhD
Senior Editor
Nature Methods

Reviewer #2 (Remarks to the Author):

The authors have fully responded to the points raised in my review. I recommend the manuscript for publication.

Version 2:

Decision Letter:

15th Sep 2026

Dear Dr Schreiber,

We greatly appreciate your patience when we check the revised version of your Brief Communication, "tangermeme: A toolkit for understanding cis-regulatory logic using deep learning models". I am pleased to inform you that this paper has now been accepted for publication in Nature Methods. The received and accepted dates will be 22nd Aug 2025 and 15th Sep 2026. This note is intended to let you know what to expect from us over the next month or so, and to let you know where to address any further questions.

Over the next few weeks, your paper will be copyedited to ensure that it conforms to Nature Methods style. Once your paper is typeset, you will receive an email with a link to choose the appropriate publishing options for your paper and our Author Services team will be in touch regarding any additional information that may be required.

If you have any questions about our publishing options, costs, Open Access requirements, or our legal forms, please contact ASJournals@springernature.com

Once proofs are generated, they will be sent to you electronically and you will be asked to send a corrected version within 48 hours. It is extremely important that you let us know now whether you will be difficult to contact over the next month. If this is the case, we ask that you send us the contact information (email, phone and fax) of someone who will be able to check the proofs and deal with any last-minute problems.

If, when you receive your proof, you cannot meet the deadline, please inform us at rjsproduction@springernature.com immediately.

If you have posted a preprint on any preprint server, please ensure that the preprint details are updated with a publication reference, including the DOI and a URL to the published version of the article on the journal website.

You may wish to make your media relations office aware of your accepted publication, in case they consider it appropriate to organize some internal or external publicity. Once your paper has been scheduled you will receive an email confirming the publication details. This is normally 3-4 working days in advance of publication. If you need additional notice of the date and time of publication, please let the production team know when you receive the proof of your article to ensure there is sufficient

time to coordinate. Further information on our embargo policies can be found here: https://www.nature.com/authors/policies/embargo.html

Authors may need to take specific actions to achieve compliance with funder and institutional open access mandates. If your research is supported by a funder that requires immediate open access (e.g. according to Plan S principles) then you should select the gold OA route, and we will direct you to the compliant route where possible. For authors selecting the subscription publication route, the journal's standard licensing terms will need to be accepted, including self-archiving policies. Those licensing terms will supersede any other terms that the author or any third party may assert apply to any version of the manuscript.

If you are active on X or Bluesky, please e-mail me your and your coauthors' handles so that we may tag you when the paper is published.

To assist our authors in disseminating their research to the broader community, our SharedIt initiative provides you with a unique shareable link that will allow anyone (with or without a subscription) to read the published article. Recipients of the link with a subscription will also be able to download and print the PDF. As soon as your article is published, you will receive an automated email with your shareable link.

Please note that you and your coauthors may order reprints and single copies of the issue containing your article through Springer Nature Limited's reprint website, which is located at http://www.nature.com/reprints/author-reprints.html. If there are any questions about reprints please send an email to author-reprints@nature.com and someone will assist you.

You can now use a single sign-on for all your accounts, view the status of all your manuscript submissions and reviews, access usage statistics for your published articles and download a record of your refereeing activity for the Nature journals.

Please feel free to contact me if you have questions about any of these points. Thank you very much again for publishing your paper with us!

Best regards,

Lin Tang, PhD
Senior Editor
Nature Methods

** Visit the Springer Nature Editorial and Publishing website at www.springernature.com/editorial-and-publishing-jobs for more information about our career opportunities. If you have any questions please click here.**

Open Access This Peer Review File is licensed under a Creative Commons Attribution 4.0 International License, which permits use, sharing, adaptation, distribution and reproduction in any medium or format, as long as you give appropriate credit to the original author(s) and the source, provide a link to the Creative Commons license, and indicate if changes were made. In cases where reviewers are anonymous, credit should be given to 'Anonymous Referee' and the source.
The images or other third party material in this Peer Review File are included in the article's Creative Commons license, unless indicated otherwise in a credit line to the material. If material is not included in the article's Creative Commons license and your intended use is not permitted by statutory regulation or exceeds the permitted use, you will need to obtain permission directly from the copyright holder.
To view a copy of this license, visit https://creativecommons.org/licenses/by/4.0/

Dear Dr. Lin Tang

Thank you for managing the submission of my manuscript. Below is my response to the reviewers, with the reviewers' comments indented and in blue, my response to them in black, and edited text from the revised manuscript in red.

> Reviewer #1 (Remarks to the Author):
>
> This manuscript introduces tangermeme, a comprehensive Python toolkit for post-training analysis of genomic deep learning models. The author addresses an important gap in the field by providing optimized, model-agnostic implementations of interpretation methods alongside novel algorithms for seqlet calling and annotation.
>
> Major Strengths
> - The focus on "everything-but-the-model" is well-motivated. The author correctly identifies that while model training tools exist, comprehensive post-training analysis frameworks are lacking.
>
> - The optimizations are impressive, one-hot encoding hg38 chr1 in under 2 seconds represents very useful and practical improvement. The built-in batching for DeepLIFT/SHAP removes memory constraints while maintaining exactness, addressing a real limitation in existing tools.
>
> - The recursive seqlet caller is a genuine contribution, being the first to call variable-length seqlets using a principled statistical framework.
>
> - The separation of sequence manipulations from model operations (Figure 1D) elegantly enables previously difficult analyses, such as computing attributions after variant substitution.

Thank you for your positive assessment of the work.

> Areas for Improvement
>
> - Overall, I believe that the manuscript would benefit from defining technical terms (e.g., marginalization, screening, convergence deltas etc) and providing brief contextual explanations to make it accessible to the broader Nature Methods readership beyond specialists in genomic deep learning. More importantly, explaining how exactly the mentioned operations serve the purpose of "distilling learned cis-regulatory patterns into insights" would improve the manuscript ie. why are these operations critical and how are they useful.

Thank you for pointing out this area of ambiguity. I have replaced the existing paragraph with two longer paragraphs that are clearer on these points.

An important consequence of this modular design is that sequence manipulations and model operations can be stacked to create useful genomic analyses (Fig 1B). *In silico* marginalizations compare model predictions before and after substituting a (usually short) sequence into a template, and thus can be used to quickly identify which motifs from a database a model responds to. Ablations are the conceptual opposite of marginalizations and compare predictions before and after shuffling a region in the input that the model may potentially be responding to. Variant effect predictions compare model predictions before and after making one or a small number of (usually) non-contiguous substitutions and can be used to fine-map candidate mutations to identify those potentially driving phenotypes. Importantly, prediction does not have to be the second step after sequence manipulation; any model operation, including DeepLIFT/SHAP, *in silico* saturation mutagenesis, or any custom operation, can be performed after the sequence manipulation in each of these settings. This functionality enables users to easily perform sophisticated genomic analyses and unlocks a new set of understudied analysis algorithms. More importantly, this modularity allows users to build custom analyses tailored for their specific research questions. The influence of surrounding context on TF binding can be easily studied by ablating the nucleotides surrounding a known motif; the suitability of an regulatory element for use in a construct can be estimated by marginalizing the entire regulatory element; teasing apart the individual and joint effects of variants at the same locus can be done in a small number of commands.

Additionally, tangermeme supports a variety of DNA design methods, with many being more thoroughly described by De Winter, Konstantakos and Aerts [31]. The simplest such method is *screening*, where sequences are randomly generated and scored by the model according to a user-defined objective function and the best sequence is returned. Greedy substitution (sometimes called "directed evolution") involves iteratively updating a randomly generated sequence by considering all possible single-nucleotide variants and choosing the single best one at each stage. Motif implantation proceeds similarly to greedy substitution except that entire motifs from a user-provided database are considered instead of individual mutations. Finally, a novel construct marginalization method is included which conceptually merges *in silico* marginalization and design. Here, the task is to create short constructs that yield specified changes in model predictions *on average* across many background sequences. This procedure can be used to design libraries of inserts for constructs that induce desired activity, e.g., certain levels of chromatin accessibility, without needing to know anything about the construct.

See my response to your later point for an amended paragraph discussing convergence deltas.

- Figure 1A's comparison table would benefit from brief definitions of each functionality category

I have expanded the caption to briefly describe each one of the categories. Modified panels from the figure itself will be shown later on in response to your specific points about it.

Figure 1: **An overview of the tangermeme software suite.** (A) A comparison of functionality divided into support for model repositories and training (Models), the implemented genomic analyses (Core Operations), support for seqlet calling and usage (Seqlets), support for biological design (Design), and built-in integration for external methods with mature code-bases (External). Orange X's indicates functionality that uses tangermeme. (B) A cartoon schematic and simulation results demontrating that sequence manipulations (the rows) can be separated by any model operation (the columns) and what expected results might be when marginalizing a motif the model responds to, ablating out a motif the model responds to, and introducing a variant that disrupts the motif. (C) Timings for one-hot encoding hg38 chr1, averaged across ten runs. Standard errors are plotted but, except for gReLU, are too small to easily see. (D) Covergence deltas from DeepLIFT/SHAP in two settings. With tangermeme, the custom profile head operation is provided to the function and so correctly registered, whereas Captum does not easily allow this.

- The convergence delta comparison (Figure 1B) is compelling but needs more explanation of why other implementations fail and why this is a critical metric.

I have significantly amended the paragraph to provide a clearer description of the problem and its consequences.

By focusing on abstract implementations of these algorithms and then rigorously validating them, we identified underappreciated issues in other commonly used codebases. DeepLIFT/SHAP is widely used for calculating feature attributions and involves overriding the backwards pass of non-linear operations to incorporate a reference sequence. However, identifying *which* operations are non-linear requires looking up each computational layer against an internal dictionary. These implementations will silently fail when encountering non-linear operations outside this internal dictionary or when these operations, usually activations, are re-used. There is usually no documentation or warning that this may happen. A user can only identify that this has happened when the convergence deltas, which theoretically should be zero (but near machine precision in practice), become significantly higher, indicating that the resulting attributions are incorrect. In contrast, tangermeme will never silently fail; instead, it will raise a warning when convergence deltas are too high, can be set to always print the convergence deltas for monitoring, and one can easily pass in custom non-linear functions to avoid this issue altogether (Fig 1D). Further documentation on this issue and how to resolve it is available alongside the rest of tangermeme's documentation.

- I think Figure 1D is extremely confusing and can be improved. It is unclear why the same "before" sequence has different profiles. It is unclear why the "before" profile of ablation is same as the "after" profile of the marginalization. Maybe what is meant is that the "before" sequences in ablation and VEP contains the blue AP-1 motif so that the implanted AP-1 is ablated and substituted? Does the ablation sequence have a meaning? It would be very useful if the caption elaborates that is the goal of these operations and how they are done. More importantly, beyond "what these operations are" the audience should be informed about "why these operations matter".

I apologize for the confusion. I have taken several steps to hopefully address the issue:

1. I have modified the cartoon sequence on the left-hand side to illustrate that the toy motif (in blue) is initially present but then ablated out (in purple), highlighting that one might want to ablate out a motif rather than a random background region, as you suggested.

2. I have split the “before” and “after” predictions from each operation to improve clarity

3. I have amended the text to elaborate more on how these operations are valuable. See my response to your previous question for the precise text.

Here is the new panel (now 1B instead of 1D due to a re-ordering of the narrative). Please see the revised paper for a higher resolution version if necessary.

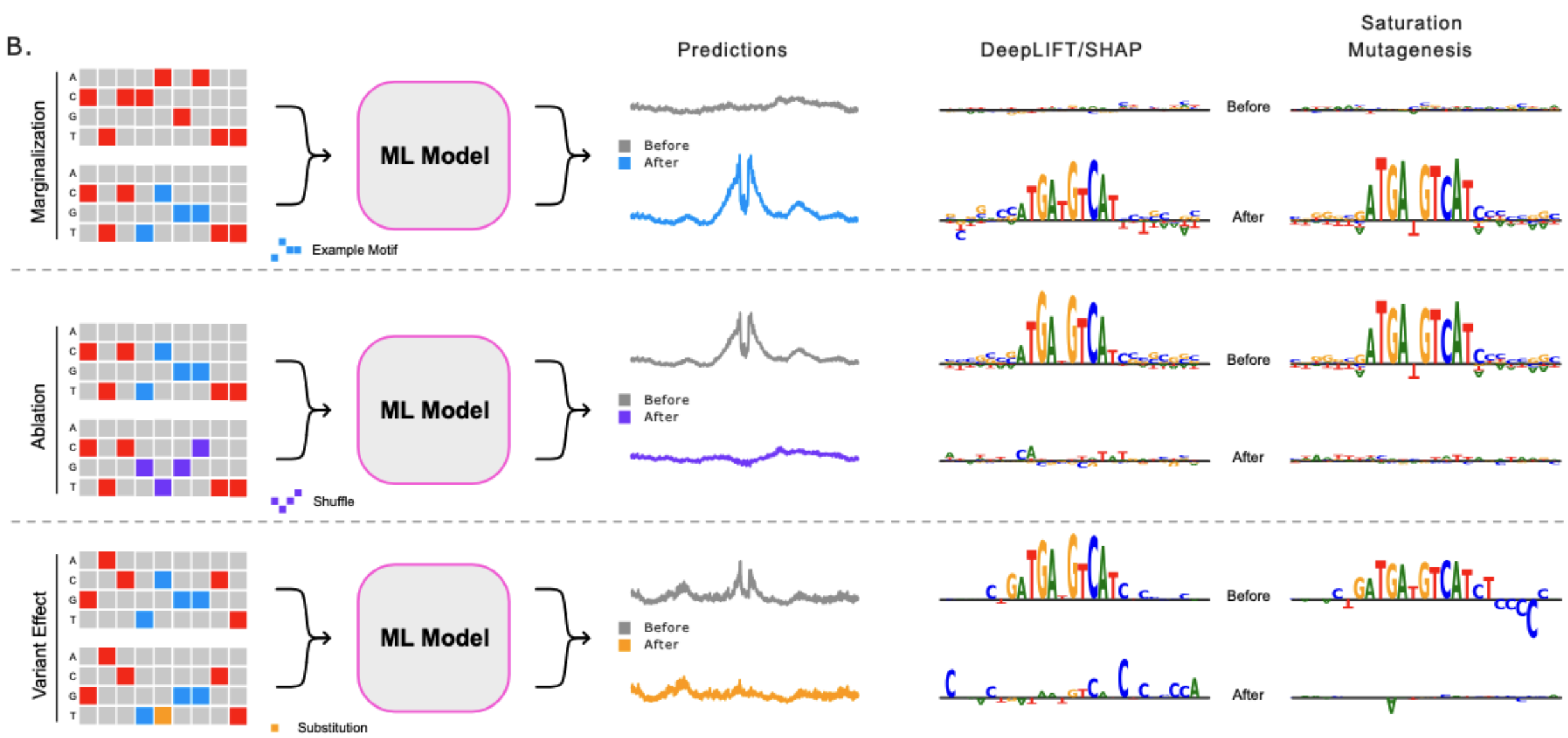

- Mentioning Kipoi et al (kipoi-interpret, kipoi-seq, kipoi-veff etc) in Figure 1 would be fair since it is (or was) capable of some of these operations, although it is not maintained any more.

Thank you for the suggestion. Kipoi has been added to the comparisons shown in Fig 1A and also referenced in the text.

A.

	Models			Core Operations											Seqlets					Design				External	
	Model Training	Data Preprocessing	Model Zoo	Sequence Manipulation	Stacked Operations	Cartesian Products	DeepLIFT/SHAP	PISA	Saturation Mutagenesis	Marginalizations	Ablations	Spacings	Variant Effect Prediction	Plotting	Seqlet Calling	Seqlet Annotation	Seqlet Ablation + Marg.	Annotation Counting	Annotation Spacing	Screening	Greedy Substitution	Motif Implantation	Construct Marginalization	Ledidi	TF-MoDISco
Selene	x								x				x	x											
kipoi	x		x				x		x				x												
EUGENe	x	x	x	x			x		x	x		x		x							x				
gReLU	x	x	x	x			x		x	x	x	x	x	x							x	x		x	x
CREsted	x	x	x	x					x					x							x	x			x
tangermeme				x	x	x	x	x	x	x	x	x	x	x	x	x	x	x	x	x	x	x	x		

- To enhance the clarity of the figures, I suggest expanding the captions for both Figure 1 and Figure 2. Specifically, for Figure 2A, the high information density makes it challenging to interpret. The caption could be improved by explicitly defining the elements, such as clarifying what the rows in the 'seqlet calling panel' represent (for instance, are they iterations of recursion?). Additionally, if important, please provide a legend or description for the gray coloring used in the subsequent panels.

In addition to the previously described steps toward improving Figure 1, I have also taken several steps to improve the clarity of Figure 2.

- I have added colorbars to several of the panels in Fig 2A and additional axis labels and titles to clarify what is being shown.

- I have added boundary lines to the first panel of Fig 2A to clarify where the called core seqlets are, and the additional flanking positions that can be added to them.

- I have expanded the caption to tie together the entire process of calling and using seqlets.

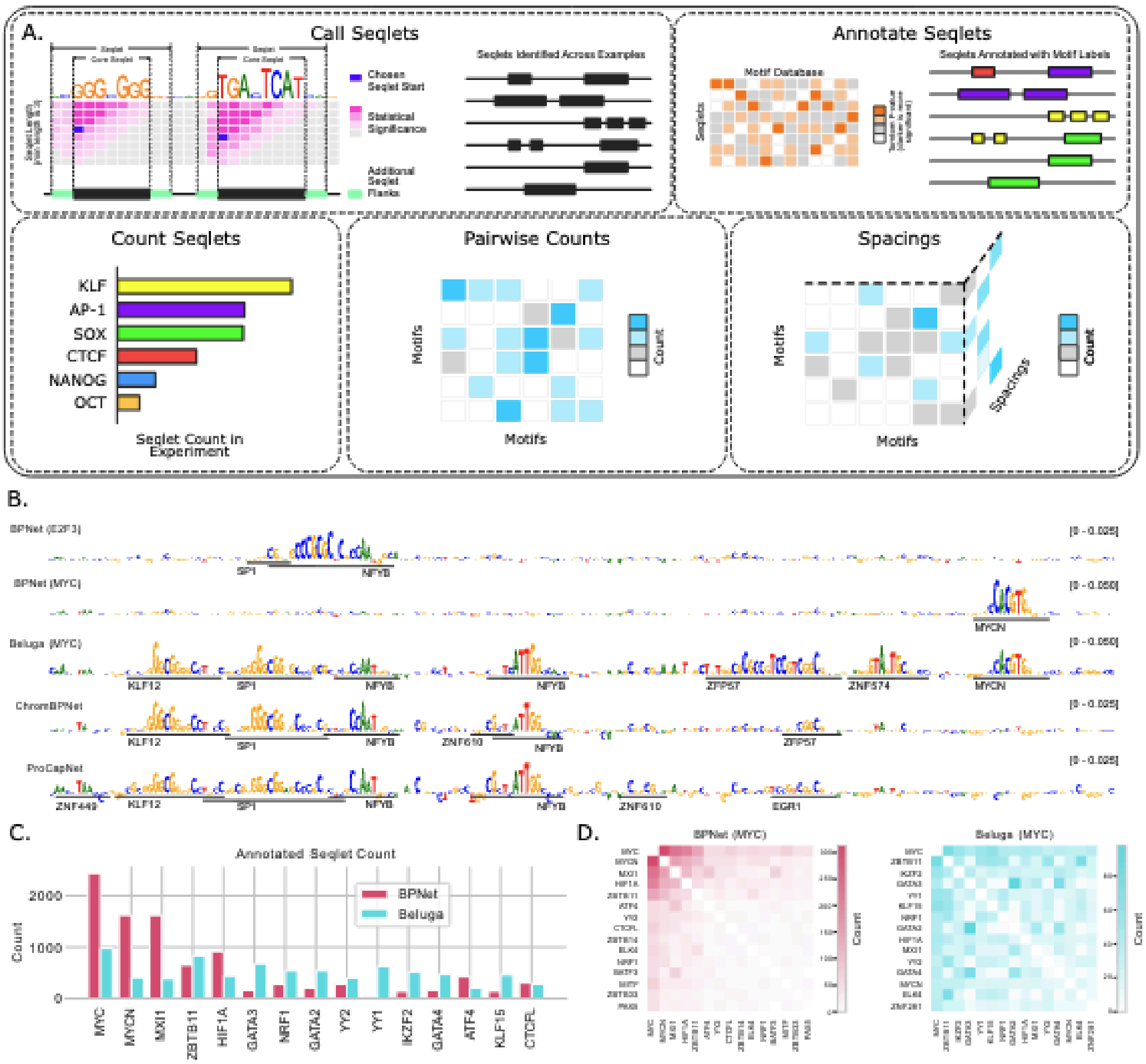

Figure 2: **An automatic pipeline for the identification and usage of seqlets.** (A) An overview of the implemented steps, beginning with the novel seqlet calling algorithm, annotation of those seqlets using a motif database, annotation counting for each motif, counting the number of pairs of each annotation and, finally, considering spacings. (B) Example attributions from several models at the PLD6 promoter along with seqlet calls (the bars underneath) and annotations. (C) Motif counts for BPNet and Beluga models predicting MYC binding. (D) Pairwise motif counts for the models in C showing the top 15 motifs for each.

- Figure 2D Beluga panel can be improved by ordering rows and columns by clustering. If there is anything biologically interesting or well-known in the pairs, it would be good to highlight that.

Thank you for the suggestion. The rows and columns are already ordered by average value, and using hierarchical clustering instead produces plots that are aesthetically less pleasing (to me at least) while not revealing anything particularly exciting. Below are versions of the figures when using hierarchical clustering. These versions split ZBTB11 and HIF1A away from the main cluster for the BPNet model, which I disagree with, and the only trend in the plot for the Beluga

model is that MYC and ZBTB11 are together, which are already the first two rows/columns in the original plot. Given that one of the points of the analysis is that the BPNet model focuses on only the MYC motif (and its variants) whereas Beluga is confounded by accessibility, I wouldn't read too deeply into the Beluga plot. Rather, it being so noisy is the point and can serve as an automated way to detect when complicated patterns are being picked up when only simple ones should be.

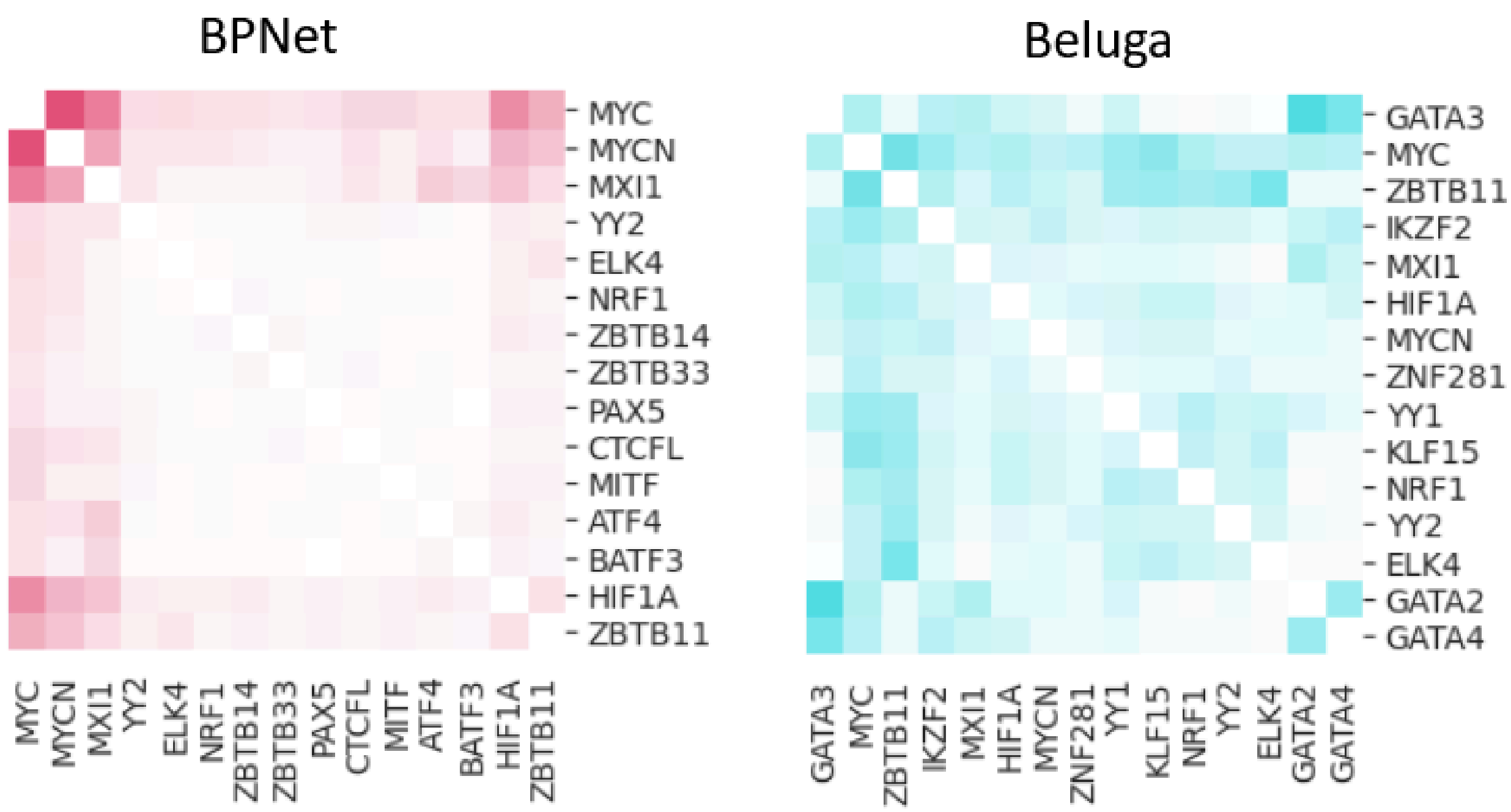

- The seqlet calling algorithm (pages 7-8) is great but mathematically dense. An intuitive explanation and/or a pseudocode before the formalism would improve accessibility.

I have added a higher-level explanation of the algorithm before the formal details.

Conceptually, the algorithm proceeds as follows. First, the attributions are binned into a user-defined number of values and the frequency of attributions falling within each bin are calculated. These frequency histograms are then used to calculate null distributions for seqlets of each potential length. These null distributions encode the probability of observing binned attribution sums of each strength by chance, assuming that attribution values are independently observed and identically distributed across positions. These nulls can then be used to directly compute p-values by summing the probability density more extreme than the observed attribution sums across the candidate seqlets. By comprehensively considering all starting positions and all seqlet lengths in a user-defined range, we can then decode the p-values to identify the longest non-overlapping seqlets that meet our criteria.

More formally, the algorithm proceeds as follows. We begin with attribution values $X \in \mathbb{R}^{n,l}$ where n is the number of examples and l is the length of these examples. In principle, l

-The demonstrations focus on TF binding and chromatin accessibility. Also mentioning that other modalities (RNA stability) can benefit from this toolkit would better showcase the toolkit's generalizability.

I have added text to the concluding paragraph addressing the recommendations.

Tangermeme is a software package for genomic deep learning that offers efficient implementations of core algorithms as well as novel algorithms for distilling learned *cis*-regulatory patterns into insights. Its operations are easy-to-use, well documented, and can be adapted or extended to research code in a variety of settings. Here, we have focused on how tangermeme can be used with models of TF binding and chromatin accessibility, but tangermeme can be used with models that make predictions for any modality, including alternative splicing, transcription, enhancer activity, and even modalities for molecules other than DNA, such as RNA stability and binding. We anticipate that tangermeme will be increasingly valuable as researchers continue to build state-of-the-art deep learning models in genomics, and that it will enable deeper analysis and biological exploration to be performed.

Recommendation
This is solid methods development work that addresses a real need in genomic deep learning. The software appears well-engineered, documented, and benchmarked. With revisions improving its accessibility for the general audience, this would be a valuable contribution to Nature Methods. The toolkit has clear potential for broad community adoption.

Thank you for your positive comments. I hope you find it helpful in future projects.

Reviewer #2 (Remarks to the Author):

Summary of key results

This work introduces tangermeme, an open-source toolkit for analyzing and interpreting sequence-to-function DNA models. The software decouples sequence manipulation (e.g., scoring genetic variants or ablating motifs) from model operations (e.g., generating predictions or feature attributions). This modular design allows researchers to "stack" these operations, such as calculating how a specific variant alters the importance scores of nearby motifs. The toolkit provides a complete workflow to uncover learned cis-regulatory logic by identifying "seqlets" (contiguous, high-importance nucleotides) from attribution scores, annotating seqlets by mapping to known TF motifs, and performing various statistical analysis of motif occurrence patterns to reveal regulatory syntax.

Originality and significance

The novelty of this work is the creation of a generalized end-to-end package for the post-training analysis and interpretation of genomic deep learning models. While existing frameworks focus on model training (e.g., Selene, EUGENe) or implement a small subset of interpretation algorithms (e.g., Captum), tangermeme provides a comprehensive solution. Additionally, the author introduces a recursive variable-length seqlet calling method.

Interestingly, this work highlights that different models (e.g. BPNet vs. Beluga) predicting the same phenomena (e.g. MYC binding) can reveal different regulatory motif logic learned by the model. This is a valuable example highlighting that attributions computed using different models can lead to different conclusions about underlying biology.

This work is presented clearly and concisely, uses appropriate statistical tests, gives appropriate credit to previous work, and provides references to data, models, and scripts necessary for reproduction.

Thank you for your positive summary of the work.

Suggested improvements and points to address:

The author states “These operations have built-in batching, support for alternative data types and devices, and work out-of-the-box on multi-input/output models” (see line 50). To improve clarity, could the authors elaborate on the practical limitations of this feature? For example, are there constraints on model architecture or block types? Could models with very long or variable-length inputs pose compatibility challenges? We suggest adding a brief note on tangermeme’s compatibility principles to clarify the expected scope of supported sequence-function models.

Thank you for pointing out additional details that could be included. Briefly, the answer is that because tangermeme is built to be so low-level, there are not constraints past what one can fit in CPU/GPU memory, and the built-in batching makes this constraint less burdensome than a quickly-written implementation might be. A more complete answer has been added into the text.

Each of tangermeme's operations are designed to simplify the practical usage of deep learning in genomics. When appropriate, operations have built-in batching, support across all data types and devices allowable in PyTorch, and work out-of-the-box on multi-input/output models. Each operation is low-level enough to be easily extended or integrated into research code bases. For instance, most DeepLIFT/SHAP implementations require all background sequences in GPU memory simultaneously, making massive models difficult to interpret. Tangermeme's built-in batching removes this requirement without compromising exactness, and low-precision support further reduces the burden. Further, tangermeme can support models with any type of internal computational units, e.g., convolutions, LSTMs, transformers, and even custom operations, and any type of prediction, e.g., a single binary value or an entire profile. These models can operate on sequences of any length, limited only by needing to have the CPU/GPU memory to run a single such sequence through the model. Finally, core operations in tangermeme are significantly faster than other software. It takes tangermeme less than two seconds to one-hot encode the entirety of hg38 chr1 (Fig 1C) — almost three times faster than the next fastest implementation. Although one-hot encoding is conceptually simple, being this fast enables on-the-fly batch generation and is reflective of the optimizations taken across tangermeme.

> Furthermore, the paper could further clarify its design philosophy for model access and integration. Is the toolkit designed for seamless integration with model repositories like Kipoi, or is the user generally responsible for loading any compatible model object into memory first?

A paragraph has been amended to more clearly state this scope. Somewhat relatedly, in response to Reviewer #1, Kipoi has been added to the comparisons in Fig 1A.

To fill this niche, we introduce tangermeme, a software package that implements "everything-but-the-model" when it comes to genomic deep learning. This design choice is intentional, as the computational operations within models and their training strategies are much more diverse and rapidly evolving than how these models are subsequently used. Instead, tangermeme focuses on making the use of these trained models as simple as possible. Accordingly, it has easy-to-use modular implementations of sequence manipulations, so as to create relevant inputs, and operations involving models, such as efficiently making predictions or calculating feature attributions (Fig 1A). This design philosophy leaves the details of loading and defining models to the user so as to not get locked in to any particular repository or the design choices made by others as to how models are stored and distributed.

> The authors report that tangermeme's one-hot encoding is more efficient and almost 3x faster than the closest best algorithm (line 61). However, the corresponding "One-hot Encodings" subsection in the Methods only describes the benchmarking procedure, not the implementation itself. Given this significant performance gain, please add a brief description of the technical optimizations or algorithmic approach.

A more thorough description of the implementation details has been included in the relevant Methods section.

Tangermeme introduces an optimized implementation of one-hot encoding a string of characters. Briefly, this approach works by avoiding unnecessary intermediate calculation and, importantly, memory allocation, which can be costly when considering long or many strings. Specifically, when encountering a string of characters, many implementations first convert the characters into numeric indices before using these indices to set the location of the ones in the final one-hot encoded matrix, requiring the allocation of an intermediary array and a two-step procedure. In our approach, we avoid this intermediary step and interpret the byte representation of the character itself as an index that allows immediate one-hot encoding without the need for an intermediate array. Tangermeme also creates a one-hot encoded representation using unsigned 8-bit integers, which is significantly faster than encoding with signed 32- or 64-bit floats. Importantly, this approach will fail gracefully when encountering unexpected characters and still allows for character sets to be ignored (usually N's) if desired.

> The authors introduce a novel algorithm to call variable-length seqlets directly from attribution scores. To validate this new method, the paper should include a quantitative benchmark against a common alternative, such as the workflow in TF-MoDISco. Key metrics to measure could include computational efficiency, identified seqlets' quality, and robustness to parameter choices.

Following your suggestion, I have included a series of comparisons between the two methods. Here is the text in the main text:

To better understand the properties of the seqlets identified by our approach, we compared them to those produced by a re-implementation of TF-MoDISco's seqlet calling agorithm (which is also included in tangermeme). As a simple synthetic dataset, we randomly generated 100 sequences, inserted the MYC motif into the center, and used DeepLIFT/SHAP to generated attributions from a MYC BPNet model using the default settings. We observed that the seqlets called using the recursive algorithm were shorter than those called using TF-MoDISco's approach, which have a long fixed default length (Supplementary Fig 1A). Although both algorithms called more seqlets than just one in the middle, TF-MoDISco covered much more of the entire example with seqlet calls than even a very permission p-value threshold (Supplementary Fig 1B). As a more quantitative task, we then considered a binary classification task where the 6 bp of the inserted sequence were positive labels and the predictions were the binary spans of seqlet calls. Interestingly, when we considered a range of p-value thresholds on this task, we observed a trend of precision and recall scores that TF-MoDISco falls near (Supplementary Fig 1C), suggesting underlying similarities in the approaches. We then timed the two algorithms on calling seqlets on an increasing number of sequences and observed that, while TF-MoDISco is faster with a very small number of sequences, that the recursive algorithm was faster for modest to large number of sequences, both in terms of examples-per-second and seqlets-per-second (Supplementary Fig 1D). An important note is that many of the design choices for the TF-MoDISco seqlet caller were made because it was part of a larger algorithm: TF-MoDISco calls wide seqlets because it can split them in subsequent steps and overcalls them because they can be easily discarded in the quality control steps. In contrast, the recursive seqlet caller is meant to be a stand-alone user-facing algorithm.

And here is the new supplementary figure.

Supplementary Figures

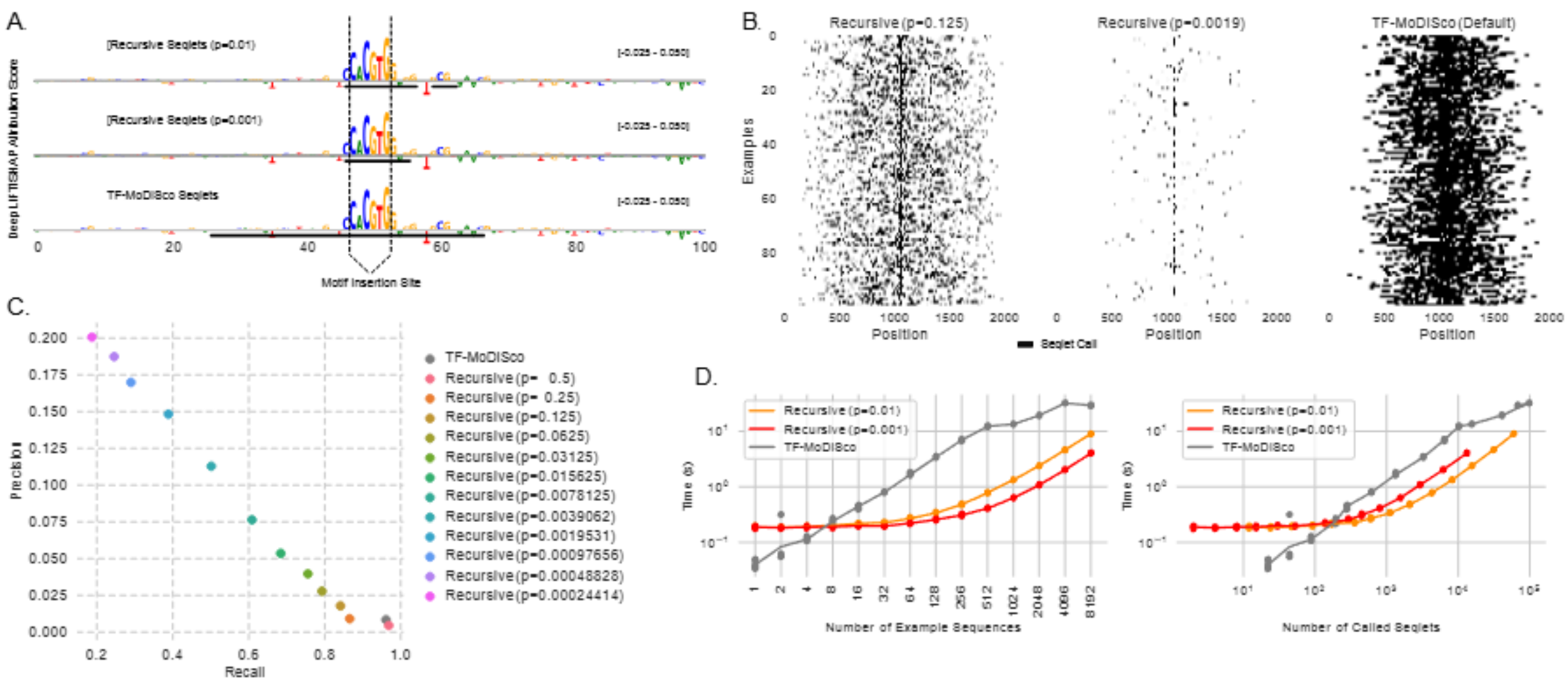

Supplementary Figure 1: **Comparison of the recursive and TF-MoDISco seqlet callers.** (A) Seqlet calls using the recursive and TF-MoDISco seqlet callers on attributions for an example sequence where a MYC motif has been inserted into the center. (B) Spans for all seqlets called at two thresholds for the recursive seqlet caller, and for the TF-MoDISco seqlet caller, across 100 examples similar to A. (C) Precision and recall values measuring whether the called seqlet boundaries identify the MYC motif inserted into the middle of the sequence and only that, across a range of p-value threshlds for the recursive seqlet caller and also the TF-MoDISco seqlet caller. (D) Timings for the recursive and TF-MoDISco seqlet callers when run 10 times per step, shown as a function of the number of examples analyzed and by the number of seqlets ultimately called.

Here is the additional text on the simulation performed:

We performed simulations to compare the seqlet caller from TF-MoDISco with the recursive seqlet caller. In these simulations, we randomly generated 100 sequences of length 2114 with uniform nucleotide probabilities and substituted the motif "CACGTG" into the center. We then used the MYC BPNet model to generate feature attribution scores for each using DeepLIFT/SHAP. This was implemented as marginalizing the MYC motif and using DeepLIFT/SHAP instead of prediction for the model operation step. We first ran the recursive seqlet caller with p-value thresholds of 0.01 and 0.001, and the TF-MoDISco algorithm with default settings, on the set of 100 sequences and plotted a representative example (Supplementary Fig 1A). We then re-ran the recursive seqlet caller with a range of p-values ranging from 0.5 to 0.5^{12} and showed seqlet boundaries across all 100 examples for the p-value threshold closest to 0.001, and a very permissive one at 0.125, and compared those to the default TF-MoDISco parameters. We then calculated precision and recall scores for each threshold by taking these masks that denoted seqlet boundaries and comparing them to labels that were set to 1 only for the central 6 bp, where the MYC motif was inserted. This comparison is imperfect in the sense that the seqlet callers would be correct if, by chance, MYC motifs occurred elsewhere in the sequence and drove model predictions in such a way that seqlets were called. However, this imperfection would penalize all seqlet calling algorithms, and does not prevent us from identifying broader trends.

Next, we timed the two algorithms when applied to a realistic seqlet calling scenario. Specifically, we calculated attributions using DeepLIFT/SHAP and the same ChromBPNet model as before on K562 ATAC-seq peaks and then used the algorithms to call seqlets. We applied the algorithms to an increasing number of examples and timed them 10 times at each step.

> The authors position tangermeme among existing toolkits like Selene and EUGENe, in a domain where wide software adoption has been challenging. What lessons from prior tools informed its design, and which key features or performance advantages are expected to overcome these adoption hurdles?

These are great questions, but their answers may be outside the scope of the paper, so I will just include them here:

(1) During my time as a core developer for scikit-learn, I learned the importance of defining the target audience for a software project and its scope ahead of time. Many projects that fail to do this well end up attempting to support too many features, become spread too thin, and eventually get abandoned before reaching their full potential. Learning from this, I specifically targeted the *developers* of genomics analysis tools as opposed to the *users* of said tools. For instance, a user of tools may wish to have a command-line tool for using ML models to calculate variant effect scores. Even though tangermeme includes variant effect predictions, implementing this command-line tool raises questions as to how to load arbitrary models, how to handle multi-task models, what distance function to use, how to handle and verify input variant files, how to report the score/scores, and how to easily install such a command-line tool on a non-expert's computer (e.g., conda environment, docker image, etc). This is a lot of work to do correctly. In contrast, tangermeme simply implements the core functionality of calculating model predictions across all tasks before and after incorporating the variants and leaves the other

details, which can vary dramatically case-by-case, to the developers. By limiting the scope in this manner and trusting my target audience, I can focus my time on adding new features. The ChromBPNet package from Anshul Kundaje's group is a good example of a project that targets the *users* of tools and not the *developers* because it is quite robust and engineered, but more challenging to build new software on top of.

(2) I use tangermeme whenever I have time to do research myself, and have incorporated tangermeme into most of my other projects. Because I am a regular user of tangermeme, I am always finding new ways to improve it and thinking about new features that would make my life easier. Further, as a new PI, I *need* it to make me more efficient because I have less time than ever to do work myself. In contrast, sometimes other packages are developed by software engineers or graduate students who then move on to other research projects or even other jobs before adoption even takes off.

> The Ledidi column in Figure 1a appears to indicate that it is incompatible with tangermeme. Is that correct?

No, it means that Ledidi is not wrapped by the tangermeme repository. I have added a paragraph elaborating on this design decision.

> For the same reason that tangermeme does not have built-in support for model repositories, it also seeks to avoid duplicating effort by wrapping other complicated algorithms when suitable implementations already exist. For example, even though Ledidi [29] and TF-MoDISco [38] have significant overlap with the design and attribution portions of the tangermeme codebase, the existing code for these algorithms is already widely used and partially being developed by others. Rather than adopting the maintenance challenge of ensuring that tangermeme is always compatible with future updates to these codebases, even when their design choices are not aligned with those in tangermeme, we instead seek to have tangermeme be compatible with *them* so as to lower the burden of their future development. This choice is similar to how there is overlap between numpy's [39] linear algebra code and scikit-learn's [40] linear models, but numpy seeks to be the foundation that accelerates development for many packages.

> Reviewer #2 (Remarks on code availability):
>
> The software and code looks very well done.

Thank you for your positive comment.

> Reviewer #3 (Remarks to the Author):
>
> I co-reviewed this manuscript with one of the reviewers who provided the listed reports. This is part of the Nature Methods initiative to facilitate training in peer review and to provide appropriate recognition for Early Career Researchers who co-review manuscripts.

Reviewer #3 (Remarks on code availability):

The GitHub repository contains an easily installable package with examples, comprehensive documentation (README and Readthedocs), and exhaustive Jupyter Notebook tutorials covering every tool described in the manuscript. I was able to install and successfully run modules of the package.

Thank you for your positive assessment of the work. I hope you find it helpful in future projects.

Jacob Schreiber
Assistant Professor
Department of Genomics and Computational Biology
UMass Chan Medical School
